# Fusion of MLP, XGBoost, and QAT-Optimized PointNet++ for Predicting Short-Term Dendrometer-Derived Stem Dynamics: An Edge-Oriented Computational Framework

**DOI:** 10.3390/s26175577

**Published:** 2026-09-02

**Authors:** Furkat Bolikulov, Kudratjon Zohirov, Gayrat Mannonov, Ulugbek Khudayorov, Zavqiddin Temirov, Ulugbek Mingboev, Erkin Hafizov, Akmalbek Abdusalomov, Young-Im Cho

**Affiliations:** 1 Department of Computer Engineering, Gachon University, Sujeong-Gu, Seongnam-Si 461-701, Gyeonggi-Do, Republic of Korea; furqat28@gachon.ac.kr; 2 Department of Software and Technical Support of Computer Systems, Karshi State Technical University, Karshi 180100, Uzbekistan; qzohirov@kstu.uz; 3 Deparment of Differential Equations, Samarkand State University Named After Sharaf Rashidov, Samarkand 140104, Uzbekistan; mannonov.g@mail.ru (G.M.); ulugbekxudayorov@gmail.com (U.K.); 4 Department of Digital Technologies, Alfraganus University, Yukori Karakamish Street 2a, Tashkent 100190, Uzbekistan; z.temirov@afu.uz; 5 Department of Computer Science and Programming, Jizzakh Branch of the National University of Uzbekistan Named After Mirzo Ulugbek, Jizzakh 130100, Uzbekistan; m_ulugbek1977@mail.ru; 6 Department of Information Systems and Technologies, Jizzakh Branch of the National University of Uzbekistan, Jizzakh 130100, Uzbekistan; h.erkin05071691@gmail.com; 7 Department of Computer Engineering, Balıkesir University, Balıkesir 10145, Türkiye; bobomirzaevich@gmail.com; 8 Department of Computer Systems, Tashkent University of Information Technologies Named After Muhammad Al-Khwarizmi, Tashkent 100200, Uzbekistan; 9 Department of Electronics and Instrumentation, Fergana State Technical University, Fergana 150100, Uzbekistan

**Keywords:** LiDAR, intelligent sensors, PointNet++, quantization-aware training, edge computing, Internet of Things (IoT), point-cloud deep learning, sensor data fusion, urban forestry, smart city monitoring

## Abstract

Urban-forest monitoring increasingly requires intelligent sensor-driven systems capable of characterizing short-term tree responses while operating efficiently within Internet of Things (IoT) and edge-computing environments. This study proposes a fusion-based artificial intelligence framework that integrates Quantization-Aware Training (QAT)-optimized PointNet++ models with machine-learning regression to predict a short-term dendrometer-derived stem-diameter response expressed in biomass-equivalent units. The framework combines 1024-point LiDAR tree representations, geometric measurements, and environmental sensor data through three components: QAT-optimized PointNet++ models for 34-species classification and trunk–crown part segmentation, frozen model-based prediction and geometric feature extraction, and MLP and XGBoost regression models for prediction of the short-term target. The dataset contained 2694 trees from five regions of South Korea, with the target derived from dendrometer-based stem-diameter measurements recorded over a 14-day interval between 8 September 2022 and 22 September 2022. Importantly, this short-term signal reflects both structural and reversible water-status-related stem dynamics and is therefore not interpreted as direct dry-biomass accumulation or carbon sequestration. The QAT-optimized models retained 92.52% segmentation accuracy (82.67% mIoU) and 80.46% species-classification accuracy, while the regression model reached R^2^ = 0.9663 and RMSE = 0.4437 kg for the defined biomass-equivalent target. Quantization reduced the saved model size of both encoders by approximately 10.5× (21 MB → 2 MB) and accelerated CPU inference by up to 4.1×. These efficiency measurements were obtained on an ×86 desktop CPU and therefore characterize computational compression benefits rather than completed deployment or field validation on a low-power embedded device. These results demonstrate the computational feasibility of combining compressed point-cloud perception with multimodal prediction of short-term dendrometer-derived stem dynamics. Validation over seasonal and multi-year periods using independent biomass-reference measurements would be required before extending the framework to long-term biomass accumulation or carbon-sequestration assessment.

## 1. Introduction

The proliferation of artificial intelligence is transforming passive environmental sensors into proactive, intelligent nodes capable of autonomous reasoning and real-time decision-making. Urban-tree monitoring includes both long-term assessment of structural biomass accumulation and short-term characterization of stem dynamics. These two objectives should be distinguished because short-interval stem-diameter variations measured by dendrometers may contain both irreversible structural growth and reversible water-status-related components. Consequently, short-term dendrometer signals cannot by themselves be interpreted as direct measurements of dry-biomass accumulation or carbon sequestration [1]. Light Detection and Ranging (LiDAR) has emerged as the sensing modality of choice, providing high-resolution 3D point clouds that can, in principle, feed directly into automated analysis pipelines [2]. The bottleneck is no longer data acquisition but the computational cost of the deep learning models required to interpret that data, a cost that is difficult to reconcile with the memory and power budgets of embedded sensors, drones, and IoT edge nodes deployed in the field. PointNet and PointNet++ have become the reference architectures for learning directly on unordered 3D point sets, using hierarchical set-abstraction layers to capture both local geometric detail and global shape [3,4]. Their accuracy comes at the price of a memory and compute footprint that is difficult to fit onto embedded hardware. Quantization-Aware Training (QAT) addresses this directly: by simulating low-precision arithmetic during training, QAT lets a network adapt its parameters to INT8 representations before deployment, typically preserving most of the full-precision accuracy while shrinking the model by 4× or more and substantially accelerating inference [5,6]. This makes QAT a natural building block for reducing the computational and storage requirements of LiDAR-based tree-analysis models and is therefore relevant to future edge-oriented implementations. In the present study, “edge-oriented” refers specifically to the computational optimization of the LiDAR-based perception pipeline through model compression and reduced ×86 CPU inference latency. It does not denote completed deployment on an embedded sensing platform. Environmental variables are supplied separately, and practical edge deployment would additionally require validation of end-to-end latency, memory use, energy consumption, sensor integration, and communication overhead on target hardware. Short-term stem dynamics are likewise not determined by geometry alone, because species identity and environmental conditions may provide complementary contextual information for interpreting dendrometer-derived responses. Multi-modal fusion, combining geometric descriptors, species-classification outputs, and environmental sensor readings, has repeatedly been shown to outperform single-modality estimators [7,8], and ensembles that pair neural networks with gradient-boosted trees capture both smooth global trends and abrupt, threshold-like effects [9]. This study develops a computationally efficient multimodal framework for predicting short-term dendrometer-derived stem dynamics from LiDAR-based structural information, predicted species identity, and environmental variables. The prediction target is derived from measurements recorded on 8 September 2022 and 22 September 2022 and is expressed in biomass-equivalent units according to the available dataset representation. Because the 14-day dendrometer signal incorporates both structural and reversible water-status-related stem-diameter variation, it is treated here as a short-term sensor-derived response rather than as a direct estimate of dry-matter accumulation.

While quantization-aware training has been shown to preserve accuracy on the perception task itself [5,6], and while separate studies have addressed point-cloud-based biomass or growth estimation with uncompressed backbones (e.g., stacked sparse autoencoders [10] or PointNeXt-based regression [11]), none of these evaluate how compression of the upstream perception model propagates to the accuracy of a downstream ecological prediction task. To the best of our knowledge, few studies have jointly examined (i) QAT-based compression of a PointNet++ perception backbone, (ii) the use of its classification and segmentation outputs in multimodal regression for a short-term dendrometer-derived stem response, and (iii) evaluation against an independently measured dendrometer-derived reference target rather than a response mathematically derived from the LiDAR inputs. This reference target represents short-term stem dynamics and is not treated as a biological reference for dry-biomass accumulation.

Accordingly, the present study does not claim to estimate dry-biomass accumulation, annual tree growth, or carbon sequestration. Its ecological target is deliberately narrower: prediction of a short-term dendrometer-derived stem response that is expressed in biomass-equivalent units in the available dataset. The principal contribution of the study is therefore methodological, namely the integration of QAT-compressed PointNet++ perception with multimodal regression for predicting this independently measured short-term response. Establishing a relationship between this response and irreversible biomass accumulation would require longer-term observations and independent biomass-reference measurements and is outside the scope of the present study.

The framework consists of independently trained PointNet++ models for tree-species classification and trunk–crown segmentation, followed by two regression models. The classification model predicts one of 34 tree species, and the predicted class is converted into a 34-dimensional one-hot vector. The segmentation model produces a trunk–crown point mask from which geometric tree measurements are obtained. These model-derived outputs are combined with environmental and vegetation-related variables to construct a 48-dimensional regression input.

The specific objectives of this study are to assess the following:

(1) Evaluate full-precision and QAT-optimized PointNet++ models for 34-class tree-species classification and trunk–crown segmentation;
(2) Construct a multimodal regression representation combining predicted species identity, geometric measurements, and environmental variables;
(3) Compare MLP and XGBoost regression models for predicting the short-term dendrometer-derived stem response over the 14-day observation interval;
(4) Quantify model-size and CPU-inference trade-offs as an initial assessment of future edge-deployment feasibility.

The principal contributions of this study are as follows:

- An integrated point-cloud processing framework that combines species classification, trunk–crown segmentation, and downstream regression of a short-term dendrometer-derived stem response;
- A 48-dimensional multimodal representation comprising 34 predicted species variables, 6 geometric variables, and 8 environmental and vegetation-related variables;
- A comparative evaluation of MLP and XGBoost regression models on a common train, validation, and test split;
- An analysis of the accuracy, model-size, and CPU-latency trade-offs introduced by QAT optimization.

The inference measurements reported in this work were obtained on an ×86 desktop CPU. Consequently, the results demonstrate computational feasibility and potential for edge-oriented deployment rather than completed validation on low-power embedded hardware.

## 2. Related Work

### 2.1. Point-Cloud Deep Learning for Forestry Applications

Deep learning has significantly improved how 3D point clouds are processed for segmentation and classification, and point clouds generated by LiDAR carry spatial information that is central to environmental monitoring, urban planning, and autonomous systems. Traditional methods such as voxelization and meshing are computationally expensive and tend to lose fine geometric detail, while deep learning models can work directly on raw point clouds. PointNet, introduced by Qi et al. in 2017, was the first model to handle unordered point sets directly, using symmetric functions such as max pooling to achieve order invariance, and it reached state of the art results on the ModelNet and ShapeNet benchmarks [3]. PointNet++ extended this work through hierarchical feature learning based on multi-scale grouping and Set Abstraction, allowing it to capture both local and global structure [4]. This made PointNet++ far more effective in complex environments such as urban landscapes and forest canopies, where point density changes drastically between the ground and the tree crown.

LiDAR itself works by measuring the distance between a laser sensor and the surface it scans, and it has become the primary sensing technology for forestry, urban planning, and environmental monitoring because of the accuracy of the spatial data it produces. At the same time, LiDAR data has its own difficulties, including sparse and uneven point density, noise, and occlusion caused by dense vegetation. Several adaptations have been proposed to address this: incorporating LiDAR-specific attributes such as elevation, reflectance, and return intensity improves a model’s ability to tell apart trunks, branches, and ground; adaptive weighting schemes give more importance to denser regions while still accounting for sparse areas; and handling multi-return LiDAR pulses helps separate individual structural layers of a tree, which matters directly for forest inventory work. More broadly, Ding et al. and Liu et al. survey the wider landscape of deep learning for 3D point-cloud processing, both confirming that noise and computational cost remain the main obstacles to reliable automated processing across domains. Li et al. proposed IPCE-Net, a dual stream architecture that fuses image-based and point-cloud-based information for farmland instance extraction and semantic segmentation, reaching 74.9 percent instance level mAP and 93.8 percent overall accuracy, which reinforces the broader point that raw sensor data benefits from careful multi-source preprocessing before a network can extract reliable structure from it [12].

### 2.2. Model Optimization and Quantization for Edge and IoT Deployment

Even though point-cloud models such as PointNet++ perform well, their computational cost makes real-time deployment on resource-constrained hardware difficult, which is a central concern for any sensor that is meant to reason locally rather than send raw data to the cloud. Quantization is one of the main tools for addressing this. Post Training Quantization converts an already trained model to lower precision and is fast to apply, but it often loses accuracy, while Quantization Aware Training simulates the effects of reduced precision during training itself, inserting fake quantization operations so the network learns weights that remain accurate once converted to INT8. Han et al. proposed Deep Compression, a three-stage pipeline combining pruning, trained quantization, and Huffman coding, achieving compression rates above 35 times on large networks such as VGG16 and ResNet without losing accuracy, an early milestone for deploying deep networks on mobile and embedded devices [13]. Wang et al. introduced HAQ, a hardware-aware automated quantization method that uses reinforcement learning to choose the best bit width for each layer based on hardware feedback such as latency and energy use, reducing latency by up to 1.95 times and energy consumption by 1.9 times [14]. More recently, Or et al. presented TorchAO, a PyTorch native framework that unifies sparsity and quantization techniques, including FP8 training, QAT, PTQ, and structured sparsity, across the full model lifecycle from training to serving, showing strong compression and speed gains on large models while keeping accuracy competitive [15].

Quantization has also been adapted specifically for point-cloud networks. Huang et al. proposed SAQAT, which adds a point importance prediction module so that quantization levels are assigned based on the semantic relevance of each point, combined with a loss function balancing classification, quantization, and prediction error; applied to PointNet, PointNet++, and DGCNN, it outperformed conventional quantization on ModelNet40 and ScanObjectNN [16]. Our own prior work applied Quantization-Aware Training directly to PointNet++ for lightweight tree classification and part segmentation, and the present study builds on and extends those results within a multimodal framework for predicting a short-term dendrometer-derived stem response [17].

### 2.3. Short-Term Stem-Dynamics Prediction and Environmental Sensing

Point-cloud-based deep learning has also been adopted quickly in ecological and environmental monitoring, particularly for forest inventory and short-term stem-dynamics prediction, where LiDAR, UAV, and multisensor data are increasingly combined. Xiang et al. proposed ForAINet, a deep learning framework for automated forest inventory from high-density airborne LiDAR, performing semantic and instance segmentation to separate stems, branches, ground, and vegetation, reaching an 85 percent F score for tree segmentation and 73 percent mean IoU across five semantic categories, and extracting biophysical parameters such as height, crown diameter, crown volume, and DBH across diverse forest types [18]. Oehmcke et al. estimated above-ground biomass directly from LiDAR point clouds using Minkowski CNNs and PointNet-based architectures, applied to Danish National Forest Inventory data, and showed that this approach can outperform traditional methods based on summary statistics while reducing preprocessing effort [19].

Combining sensing modalities has also proven valuable. Liu et al. used PointNet++ with backpack laser scanning data to classify tree species, studying how tree height, leaf and wood separation, and different downsampling strategies affect accuracy, and found that 2048 to 5120 points gave the best classification results, supporting the case for cost-effective mobile laser scanning in forest surveys [20]. Terryn et al. combined Terrestrial Laser Scanning with UAV-based laser scanning for mapping tropical rainforest structure, showing that TLS is strong at capturing individual tree metrics but struggles with occlusion in dense canopies, while UAV-LS covers larger areas and captures canopy structure well but tends to underestimate trunk diameter, so combining the two improves overall accuracy [21]. Huang et al. proposed a similar fusion strategy combining UAS-based digital aerial photogrammetry with LiDAR for short-term stem-dynamics prediction in mountainous terrain, where photogrammetry compensates for LiDAR’s terrain following limitations and LiDAR improves tree height extraction, together improving canopy height models and biomass estimates while reducing survey cost [7]. Across these studies, the consistent finding is that geometric structure from a single sensor is rarely enough on its own, and that combining sensing sources together with species and environmental information gives more reliable prediction of short-term dendrometer-derived stem responses, the same principle our fusion framework follows.

### 2.4. Fusion Architectures and Ensemble Learning

Multi-task learning offers a useful framework for models that need to perform several related jobs, such as classification, segmentation, and regression, from the same underlying sensor data. Crawshaw’s survey of multi-task learning shows that sharing internal representations across tasks improves data efficiency, reduces overfitting, and speeds up convergence, and organizes existing approaches into architectural, optimization-based, and relationship learning strategies [22]. Vandenhende et al. extended this to dense prediction tasks in computer vision and found that properly designed multi-task networks not only outperform single-task models but also reduce memory and computation, which matters directly for models meant to run on constrained edge hardware [23].

On the feature fusion side, Dai et al. proposed attentional feature fusion, using a multi-scale channel attention module to combine features from different network depths, and showed that this kind of learned attention consistently works better than simple concatenation or summation, particularly when the features being combined differ in semantic level or spatial resolution, which is relevant when combining point-cloud features with categorical species information and continuous environmental readings [24]. On the decision level, Zhu et al. showed that combining neural networks with gradient boosted trees such as XGBoost produces more accurate predictions than either method alone, since neural networks tend to capture smooth global trends while gradient boosting handles outliers and threshold-like effects more effectively, improving both accuracy and stability across different data distributions [9]. Cui et al. reviewed fusion methods that combine image and point-cloud data more broadly, organizing them by fusion level, early, mid, or late, and showed that multi-level fusion strategies tend to perform best across perception tasks such as object detection and semantic segmentation [25]. Building on these studies, the present framework uses separately trained and frozen PointNet++ classification and segmentation models as upstream prediction modules. The classification model provides a predicted species class, while the segmentation model provides a trunk–crown point mask used for geometric measurement extraction. These outputs are combined with environmental variables to form a tabular multimodal representation. MLP and XGBoost are then trained independently on the same fused input and evaluated as alternative regression models. Therefore, the proposed approach is a multi-stage, multi-model framework rather than a jointly optimized multi-task network or a decision-level ensemble.

## 3. Materials and Methods

The proposed methodology consists of five consecutive stages: point-cloud preprocessing, tree-species classification, trunk–crown segmentation, QAT optimization, and multimodal regression of the short-term dendrometer-derived stem response. Each tree point cloud is standardized to 1024 points and processed by independently trained PointNet++ classification and segmentation models. After QAT optimization, both models are frozen and used to generate predicted species and segmentation outputs. These outputs are combined with geometric and environmental variables to construct a 48-dimensional feature vector. MLP and XGBoost models are then trained independently to predict the defined short-term dendrometer-derived response over the 14-day observation interval. Model performance is evaluated in terms of classification accuracy, segmentation accuracy and mIoU, regression error, model size, and CPU inference latency [21].

### 3.1. Sensing Pipeline and Dataset

Airborne LiDAR point clouds were available for 2694 individual urban trees collected from five regions of South Korea: Daegu, Wonju, Dongtan, Sejong, and Jeju. The dataset contained paired measurements associated with two observation dates: 8 September 2022 and 22 September 2022. The interval between the two dates was 14 days. Accordingly, the prediction target used in this study is a short-term dendrometer-derived stem response measured over the 14-day observation interval. The source measurements are stem-diameter observations obtained independently from the LiDAR acquisition and PointNet++ processing pipeline. In the available dataset, these measurements are represented in biomass-equivalent units; however, because short-term stem-diameter variation may contain both irreversible structural growth and reversible water-status-related components, the resulting target is not treated as direct dry-biomass accumulation. Throughout the revised manuscript, it is therefore interpreted as a short-term stem-diameter-derived response expressed in biomass-equivalent units.

For tree i, the short-term biomass-equivalent target change was defined as

(1) $${∆B}_{i}=B_{i,t_{2}}-B_{i,t_{1}}$$

where the two terms denote the dendrometer-derived biomass-equivalent values associated with 8 September 2022 and 22 September 2022, respectively. Their difference constitutes the short-term prediction target used in this study and is expressed in kilograms according to the dataset representation. This unit should not be interpreted as evidence that the measured 14-day difference corresponds exclusively to irreversible dry-biomass accumulation. Accordingly, the biomass-equivalent transformation is treated only as the numerical representation supplied by the dataset; it does not constitute an independent biological validation of biomass gain. The modeled quantity in this study is therefore the short-term dendrometer-derived response itself, not dry-matter accumulation.

The dataset was divided into 2155 training samples, 269 validation samples, and 270 held-out test samples. All information associated with the same individual tree was treated as a single paired record to prevent observations from the two dates being distributed across different dataset partitions.

**Figure 1:**
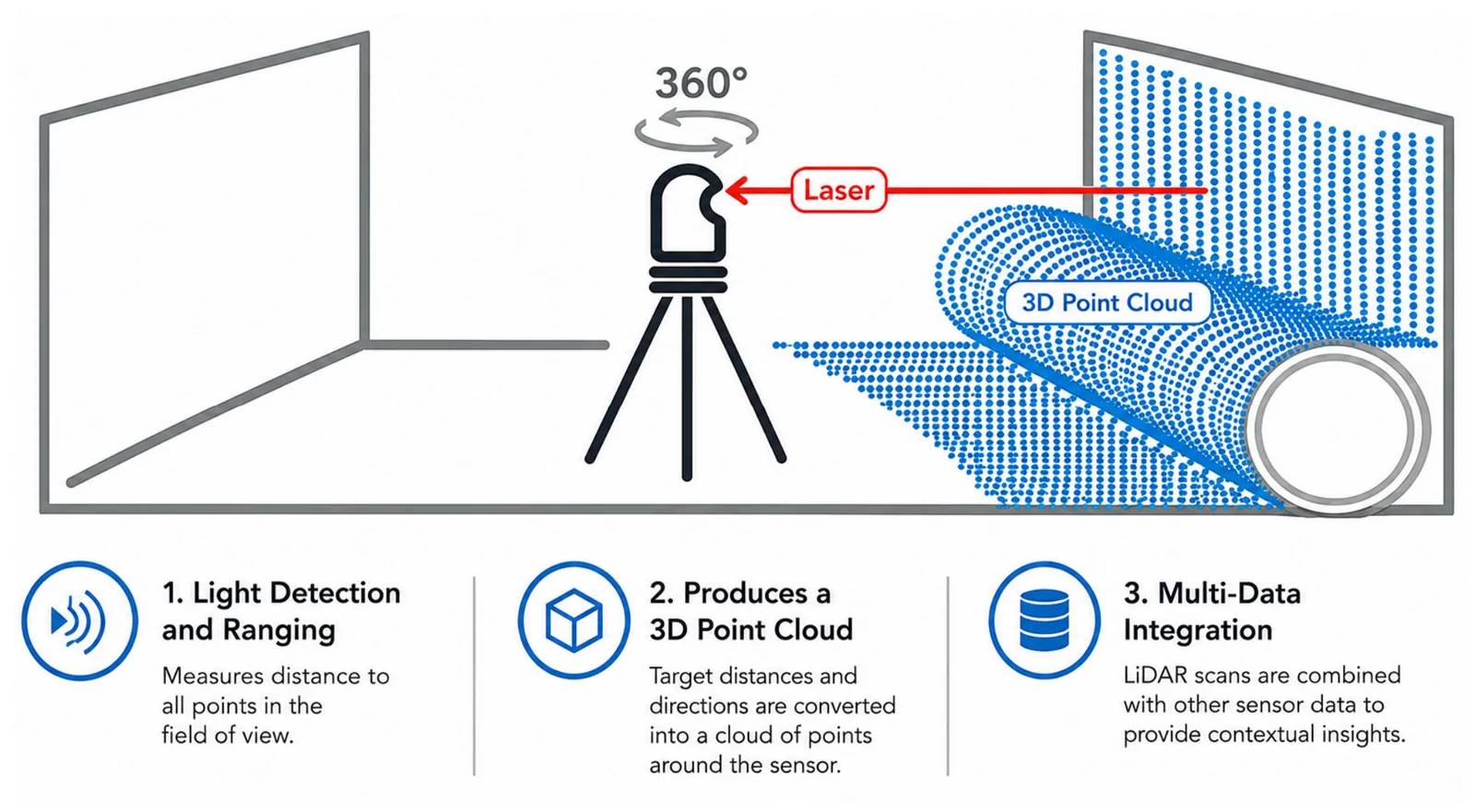
LiDAR scanning process [20].

The general scanning process, including how the sensor emits and receives laser pulses to build up a 3D point cloud of the tree canopy, is illustrated in Figure 1.

**Figure 2:**
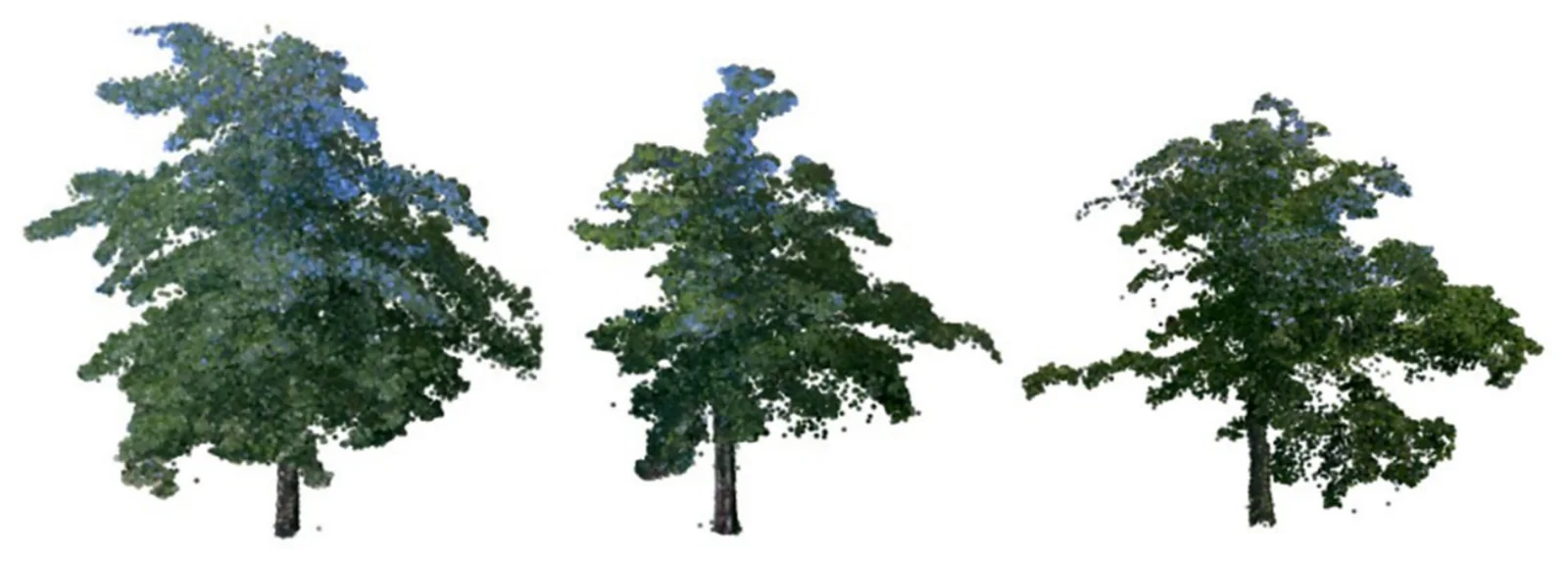
LiDAR data visualization (full points).

This figure shows the physical LiDAR acquisition setup and how laser returns from the tree canopy and ground are converted into a 3D point cloud. Raw scans differ substantially in point density depending on flight altitude, canopy density, and occlusion from neighboring trees, with an original scan containing on the order of hundreds of thousands of points per tree. Because PointNet++ expects a fixed size input, each tree was resampled to a standardized representation of 1024 points using Farthest Point Sampling, an algorithm that iteratively selects points to maximize the minimum distance from already-selected points, which keeps the resulting subsample spatially uniform rather than clustered in whichever region happened to be scanned most densely. Each retained point carries six channels: three spatial coordinates (X, Y, Z) and three RGB color values. The 1024-point representation was selected as a computational compromise between geometric detail retention and inference cost. Figure 2 shows a representative example.

This figure shows a raw, full-density point cloud of a single tree before any downsampling.

**Figure 3:**
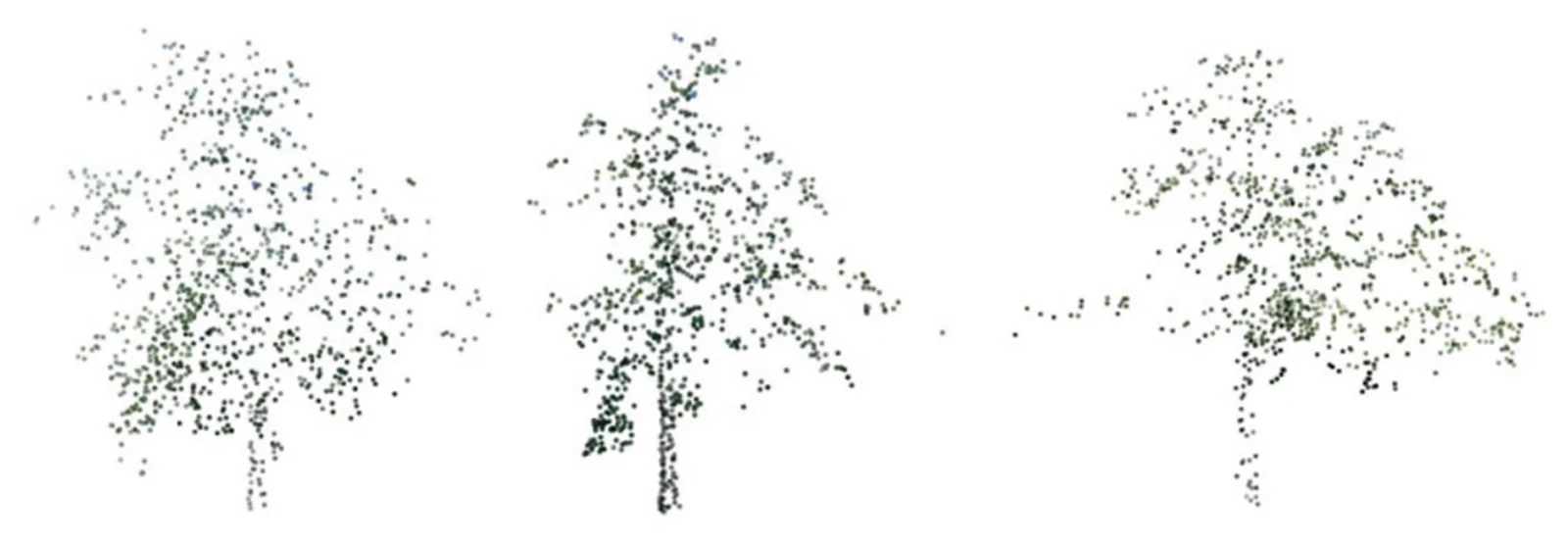
LiDAR data visualization (H5, 1024 points).

Figure 3 shows the same tree after resampling to the standardized 1024-point input used by the network, stored in the project’s HDF5 dataset format.

In addition to the point-cloud representation, each tree record included geometric, environmental, soil, and vegetation-related variables. The regression input contained six geometric variables associated with tree structure and eight auxiliary variables describing climate, soil, and vegetation conditions. The environmental group comprised temperature, CO_2_, and humidity; the soil group comprised pH and electrical conductivity; and the vegetation-related group comprised NDVI, LAI, and size. These variables were combined with the predicted tree-species representation to construct the multimodal regression input described in Section 3.4.

All continuous variables were normalized using parameters estimated from the training partition. The same fitted transformations were subsequently applied to the validation and test partitions to prevent preprocessing-related information leakage.

### 3.2. PointNet++ Architectures for Classification and Segmentation

Two independently trained PointNet++ models were used: one for tree-species classification and one for trunk–crown part segmentation. The models used an identical Set Abstraction backbone configuration but did not share trained weights. The term “common backbone” therefore refers to architectural similarity rather than parameter sharing between the two tasks [1,2]. Reusing the same backbone design across both tasks, rather than designing two unrelated networks, keeps the overall pipeline simpler to train, quantize, and maintain, and lets the same intuition about the network’s behavior transfer between tasks. Table 1 below lays out the complete configuration for both models side by side: the shared encoder stages common to both, followed by the classification-specific head and the segmentation-specific head.

The shared encoder processes the 1024-point input through three Set Abstraction (SA) stages. At each stage the number of points is reduced (1024 → 512 → 128 → 32) while the number of feature channels describing each remaining point grows (6 → 128 → 256 → 512). The radius used to group neighboring points also grows at each stage (r = 0.2 m, then 0.4 m, then 0.8 m), so that early layers process small local neighborhoods and learn local geometric and radiometric patterns, whereas deeper layers use larger receptive fields to capture branching structure, crown organization, and global tree shape. This progression loosely mirrors how a person might identify a tree species or judge its structure in the field: first noticing local texture, then branch arrangement, and only at the end forming a judgment based on the whole tree.

**Figure 4:**
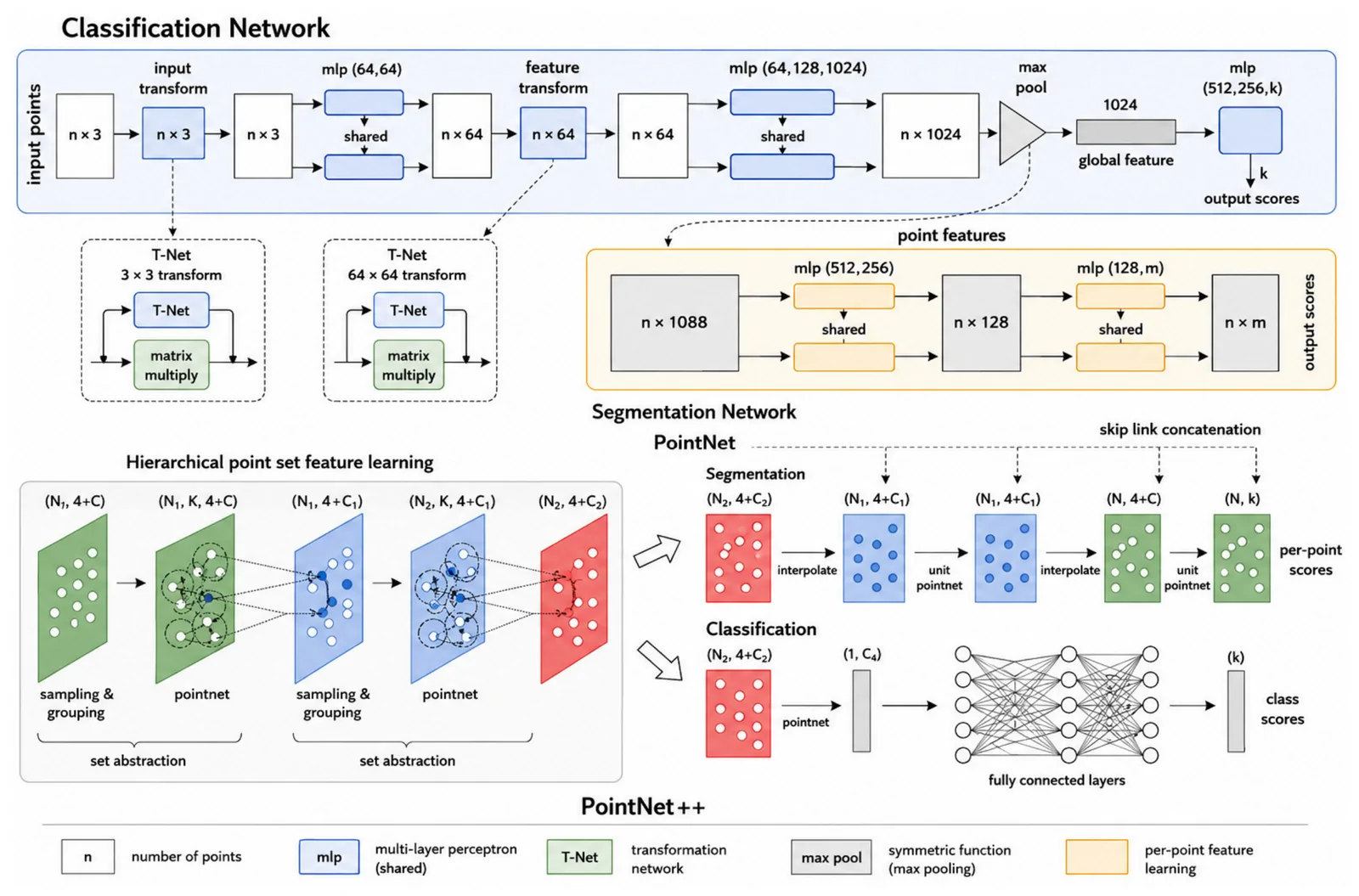
PointNet and PointNet++ architectures, showing the classification model and the part segmentation model, both built on the shared Set Abstraction backbone [3,4].

From this shared 32-point, 512-channel representation, the two tasks diverge. The classification head applies one further Set Abstraction stage with global pooling, collapsing everything down to a single 1024-dimensional descriptor for the whole tree, which is then passed through three fully connected layers with batch normalization, ReLU activation, and dropout (*p* = 0.5) to reduce overfitting [21], ending in a softmax layer over the 34 possible species. The segmentation head instead keeps the 32-point spatial structure intact and works in reverse, upsampling back to the full 1024 points through three Feature Propagation (FP) stages. Each FP stage estimates the feature at a new, denser set of points by interpolating from its nearest neighbors in the sparser previous stage, and then merges this interpolated information with a skip connection carrying features from the matching encoder stage, so both coarse semantic information (this region is probably trunk versus crown) and fine spatial detail (exact boundary location) are available when the final per-point label is predicted. These skip connections are not a minor implementation detail: without them, the segmentation model would carry strong semantic information but lose the precise spatial location needed for clean trunk–crown boundaries, since that spatial precision is exactly what repeated downsampling destroys. This encoder-decoder pattern, combining abstraction with multi-resolution fusion, recurs across many dense prediction tasks in computer vision [17], and here it is what enables trunk–crown separation for subsequent sparse-cloud structural feature extraction. Both architectures are illustrated in Figure 4.

The shared encoder configuration used by both heads is summarized in Table 1.

The classification head converts its final layer output into per-species probabilities using the standard softmax function:

(2) $$P(y=\left.j\right|x)=\frac{exp(z_{j})}{\sum_{k=1}^{34}exp(z_{k})}$$

where z_j_ is the j-th logit score. Predicted species = argmax of probabilities. The segmentation decoder’s Feature Propagation stages use inverse-distance-weighted interpolation to estimate features at each upsampled point:

(3) $$f_{interp}(x)=\frac{\sum_{i=1}^{3}\omega_{i}(x)\cdot f_{i}}{\sum_{i=1}^{3}\omega_{i}(x)},\;\omega_{i}(x)=\frac{1}{{\left\|x-x_{i}\right\|}_{2}^{2}}$$

This is the inverse-distance-weighted (IDW) interpolation formula used in each Feature Propagation stage to estimate a feature value at a new point based on its nearest neighbors in the coarser point set.

### 3.3. Quantization-Aware Training for Edge-Oriented Computational Optimization

After full-precision training, supported convolutional and fully connected layers in both PointNet++ models were optimized using Quantization-Aware Training. During QAT, fake-quantization operations simulated reduced-precision weights and activations while gradient-based optimization remained in floating point. Following fine-tuning, supported layers were converted for INT8 execution using the PyTorch fbgemm backend included in PyTorch 2.5.1 for ×86 CPUs. Geometric operations specific to PointNet++, including Farthest Point Sampling, neighborhood search, and inverse-distance interpolation, were not assumed to execute entirely in INT8. The resulting implementation should therefore be described as a QAT-optimized mixed-precision model rather than a completely integer-only pipeline [9,10]. This has been shown repeatedly to preserve accuracy substantially better than post hoc conversion [22,23]. Each quantized model was fine-tuned for 400 epochs with a batch size of 8 on CPU, rather than GPU, using the fbgemm backend included in PyTorch 2.5.1, which is optimized for INT8 matrix multiplication on standard ×86 CPUs [7]. Table 2 summarizes the exact quantization configuration used for both encoders.

**Figure 5:**
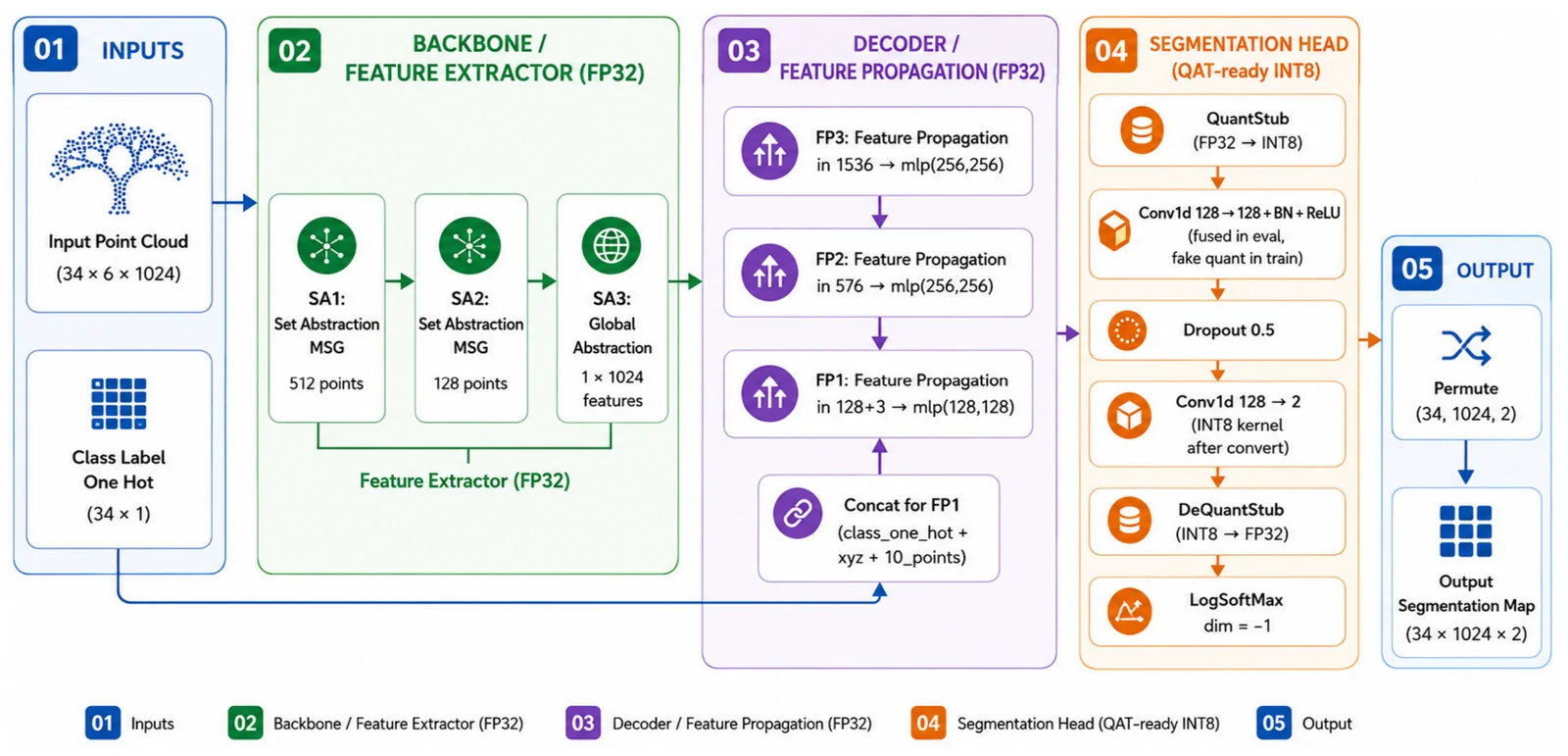
Simplified QAT-ready PointNet++ segmentation architecture, showing the input point cloud and class label, FP32 backbone and feature-propagation decoder, INT8 quantization-aware segmentation head, and final per-point segmentation output.

The figure below illustrates the resulting quantized architecture, showing where fake-quantization and dequantization operations are inserted relative to the original FP32 design in Figure 4; the resulting architecture is shown in Figure 5, where the underlying set-abstraction and feature-propagation structure is otherwise unchanged, only the numerical precision at each layer differs.

### 3.4. Multimodal Feature Construction and Regression

After QAT optimization, the classification and segmentation models were frozen and used as upstream prediction modules. Their parameters were not updated during regression-model training.

For each tree, the classification model generated a predicted species label:

(4) $$\hat{z_{i}}=arg\;\underset{c}{\max}\;P(\left\langle c|X_{i}\right\rangle )$$

where $X_{i}$ denotes the tree point cloud and c represents one of 34 species classes. The predicted species label was converted into a 34-dimensional one-hot vector:

(5) $$s_{i}\in R^{34}$$

The segmentation model generated a two-class trunk–crown mask. Six geometric variables associated with tree structure were then computed directly from the same 1024-point, PointNet++-segmented point cloud used as the network’s input: A sparse-cloud DBH-related descriptor was derived from segmented trunk points using the maximum horizontal pairwise point-to-point distance within a narrow vertical region around breast height (1.3 m). Because the 1024-point representation distributes a limited point budget across the entire tree, only a small subset of points may be available near breast height. Accordingly, this quantity is treated as a coarse structural descriptor rather than as a field-equivalent DBH measurement. Crown width (E–W, N–S) was computed as the coordinate range of the segmented crown points along each horizontal axis, crown height was the vertical extent of the crown points, and crown density was computed as the ratio of crown point count to convex-hull volume. Because this sparse-cloud DBH proxy relies on a comparatively small number of points within the breast-height band, it is expected to be substantially more sensitive to sampling density, segmentation error, and isolated trunk/branch points than crown-derived variables. Accordingly, it is used here only as one structural input to the multimodal regression model and is not presented as a validated replacement for conventional field DBH measurement. A post hoc point-density sensitivity check further confirmed that this DBH-related descriptor is substantially more sensitive to sparse sampling than the crown-derived variables. For example, within a ±0.05 m diagnostic band around breast height, the median 1024-point tree contained only five segmented trunk points in the band, and 17.9% of trees contained fewer than two points, preventing pairwise diameter calculation. Wider bands increased point availability but also increased sensitivity to non-stem and isolated points. These observations motivate the conservative treatment of this variable as a sparse-cloud structural proxy rather than a validated DBH measurement. The sensitivity analysis results are summarized in Table 3.

Eight additional variables represented environmental, soil, and vegetation conditions: temperature, CO_2_, humidity, soil pH, electrical conductivity, NDVI, LAI, and size. After normalization, the three feature groups were concatenated:

(6) $$x_{i}=[s_{i};\;g_{i};\;e_{i}]\in R^{48}$$

where $s_{i}\in R^{34}$ is the predicted species one-hot vector, $g_{i}\in R^{6}$ contains the geometric variables, and $e_{i}\in R^{8}$ contains environmental and vegetation-related variables.

**Figure 6:**
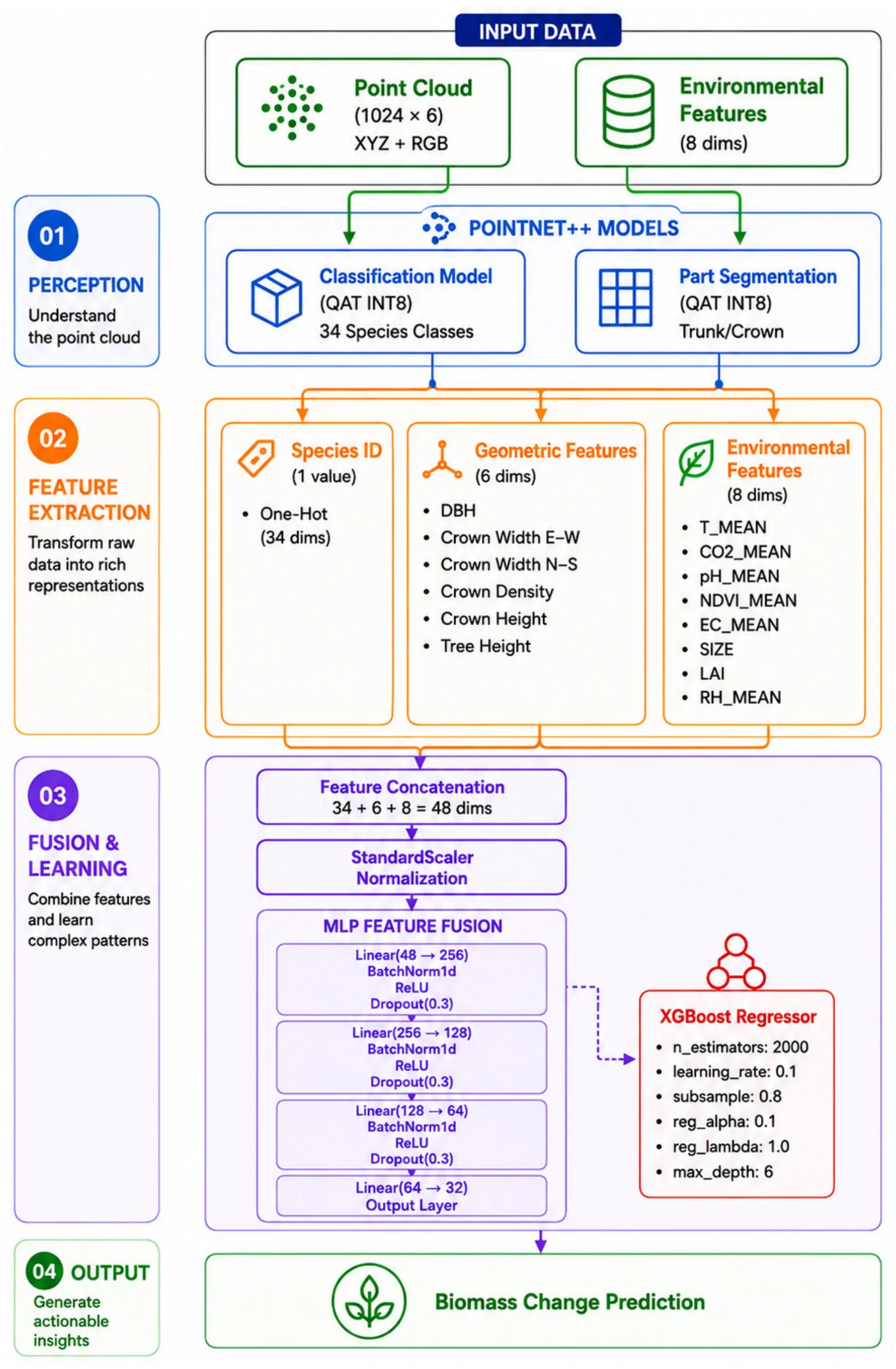
Proposed multimodal regression pipeline. The frozen classification model generates a predicted species class represented as a 34-dimensional one-hot vector. The segmentation output supports the extraction or inclusion of six geometric variables. These features are combined with eight environmental and vegetation-related variables to form a 48-dimensional input for independently trained MLP and XGBoost regression models.

Two independent regression models were trained using the same 48-dimensional input. The first was an MLP with hidden layers of 256, 128, and 64 units followed by a scalar regression output. The second was an XGBoost regressor with 200 estimators, maximum depth of 6, learning rate of 0.1, subsample ratio of 0.8, and column-sampling ratio of 0.8. The models were evaluated separately. No weighted averaging, stacking, or decision-level fusion of their predictions was applied. To directly evaluate whether the high regression performance could be explained by a simple current-size effect, additional ordinary least-squares linear regression baselines were fitted using DBH alone, tree height alone, and DBH together with tree height. These diagnostic models used the same 2155-tree training partition and the same 270-tree held-out test partition as the primary regression models. The purpose of these baselines was not to provide alternative ecological models, but to directly test whether the short-term prediction target exhibited the approximately linear relationship with baseline tree size suggested by the Reviewer. Pearson and Spearman correlations between the target and the individual DBH and tree-height variables were also calculated. R^2^ was used as the primary diagnostic metric for these simple structural baselines, while Pearson and Spearman correlations were used to assess linear and monotonic associations with the short-term target. The full pipeline is illustrated in Figure 6.

Table 4 breaks this 48-dimensional vector down two ways at once: by which model or sensor produced each group of features, and by what physical category those features represent ecologically. Species identity alone accounts for 34 of the 48 dimensions, which foreshadows a result confirmed later in Section 4.4, where species-related features show the strongest association with the predicted short-term dendrometer-derived response among the evaluated feature groups. Combined with the six geometric features described above, 40 of the 48 input dimensions (83%) are therefore generated entirely by the LiDAR-PointNet++ pipeline itself, without any separate high-density scan or manual field measurement. Only the remaining eight dimensions require external environmental or vegetation-related measurements. Consequently, the present results should be understood as an evaluation of computationally compressed LiDAR perception and multimodal prediction, rather than as validation of a fully integrated edge sensing device.

With the fused feature vector defined, two parallel regression heads are trained on it, chosen specifically because they make errors in different, complementary ways. Table 5 summarizes both.

The MLP maps the 48-dimensional fused input through hidden layers of 256, 128, and 64 units before producing the final short-term dendrometer-derived response prediction. The XGBoost head, trained on the same fused feature vector, instead provides a tree-based alternative that can capture nonlinear interactions and threshold-like relationships in the short-term dendrometer-derived target [19,26]. The two regression models were evaluated independently to compare neural-network and gradient-boosting approaches under the same feature representation and dataset split.

**Figure 7:**
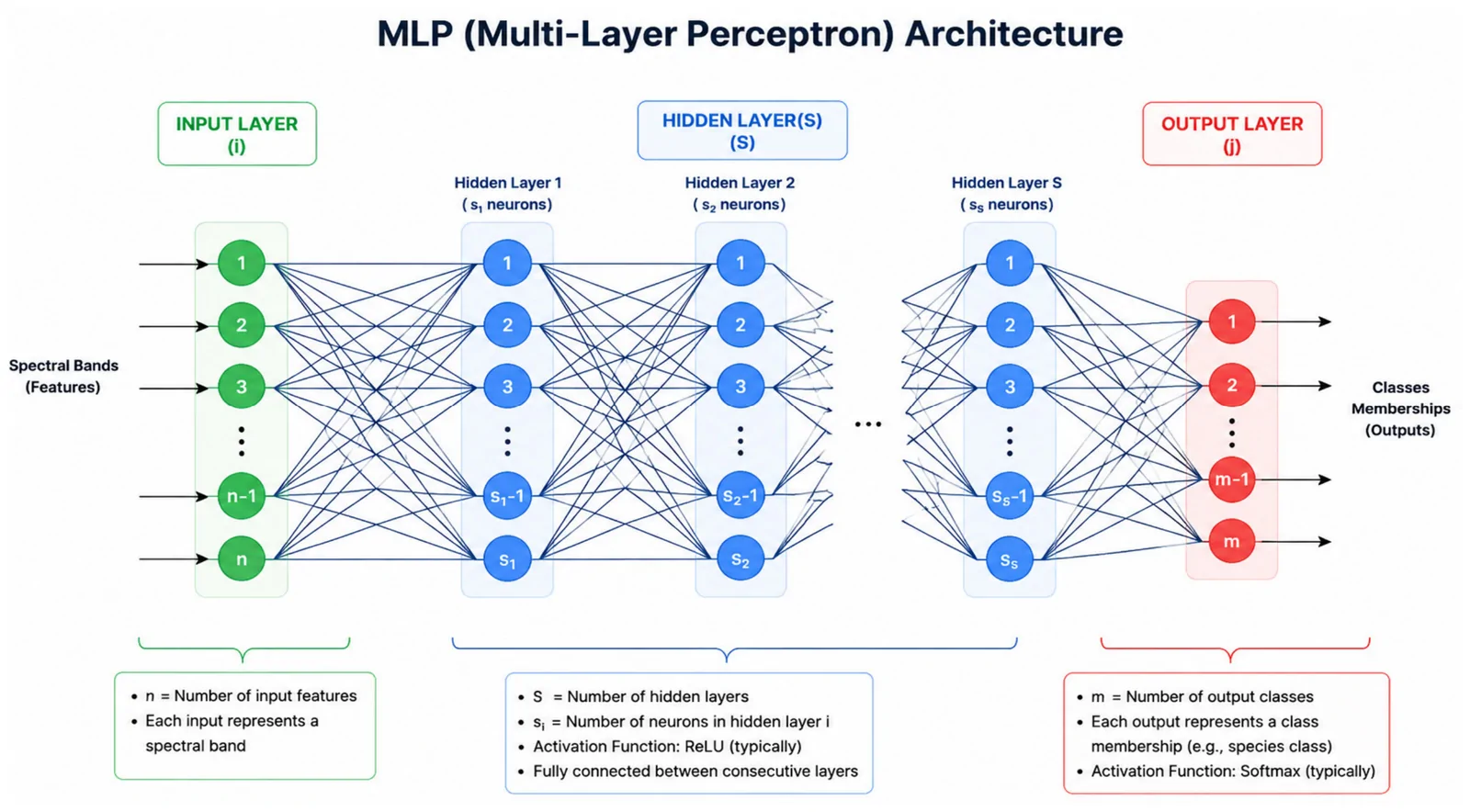
MLP (Multi-Layer Perceptron) architecture [27].

**Figure 8:**
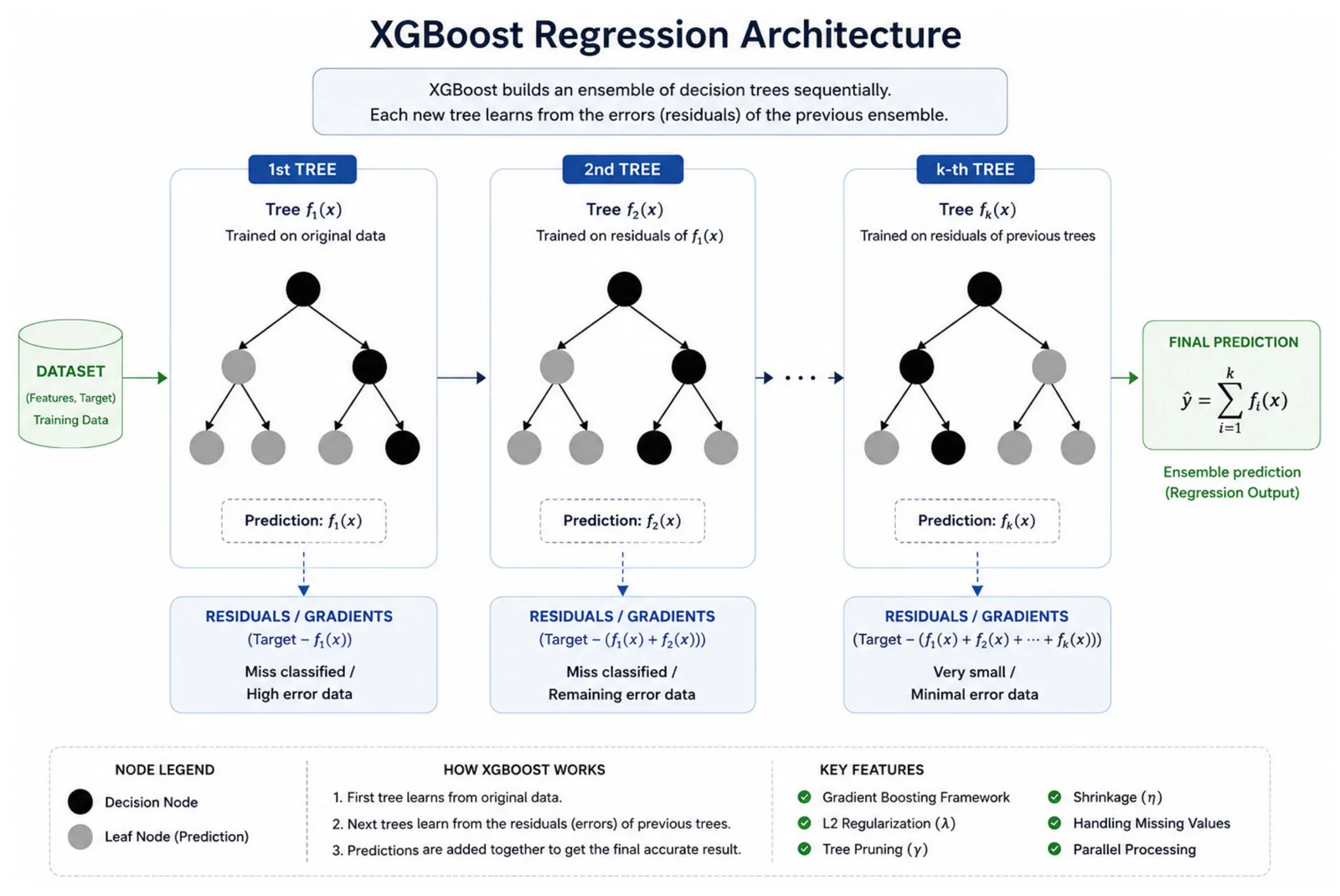
XGBoost regression architecture, showing the gradient-boosted ensemble structure of the second regression head [23].

Figure 7 shows the three hidden layers and output layer of the MLP regression head. Figure 8 illustrates the corresponding XGBoost ensemble structure.

### 3.5. Evaluation Metrics

To assess performance consistently across the classification, segmentation, and regression stages of the pipeline, we use a set of standard metrics from the computer vision and machine learning literature, each chosen for a specific reason explained below.

For the 34-class species classification task, overall accuracy (also called instance accuracy) is the proportion of correctly classified trees among all test samples:

(7) $$Accuracy=\frac{Number\;of\;Correct\;Predictions}{Total\;Number\;of\;Predictions}=\frac{1}{N}\sum_{i=1}^{N}1(y_{i}={\hat{y}}_{i})$$

This metric provides a global view of classification performance but can be misleading for imbalanced datasets where a model might achieve high accuracy by predicting only the majority class.

Mean Class Accuracy

The average of per-class accuracies, computed as

(8) $$Mean\;Class\;Accuracy=\frac{1}{C}\sum_{c=1}^{C}\frac{TP_{c}}{TP_{c}+FN_{c}}$$

where C is the number of classes (34 species), $TP_{c}$ is the number of true positives for class c, and $FN_{c}$ is the number of false negatives. This metric treats all classes equally, regardless of their frequency, providing a more balanced assessment for imbalanced datasets. Additionally, the confidence score (i.e., the predicted probability of the chosen class obtained from the softmax layer) is reported for classification samples.

Confidence represents the probability score assigned by the model to the predicted class, obtained via the softmax function:

(9) $$Confidence({\hat{y}}_{i})=\underset{c\in C}{\max}\frac{e^{z_{c}}}{\sum_{j=1}^{\left|C\right|}e^{z_{j}}}$$

where $z_{c}$ is the logit for class c, and C is the set of all classes.

Although confidence is not considered a standard evaluation metric, it is commonly used to provide insights into model certainty and prediction reliability. Recent works have leveraged confidence estimation for uncertainty analysis and active learning in 3D vision tasks. For segmentation, three evaluation criteria are reported: accuracy, which measures the overall proportion of correctly labeled points across the entire point cloud.

It calculates the proportion of correctly predicted point labels across the whole point cloud:

(10) $$Overall\;Accuracy=\frac{\sum_{i=1}^{N}1(y_{i}={\hat{y}}_{i})}{N}$$

where N is the number of points, ${\hat{y}}_{i}$ is the predicted label for point i, and $y_{i}$ is the ground-truth label. Per-class Intersection over Union (IoU) computes the overlap between predicted and ground-truth points for each semantic part (e.g., trunk and crown). It measures the overlap between predicted and ground-truth labels for each semantic part (e.g., trunk, crown):

(11) $$IoU_{k}=\frac{\left|P_{k}{\cap G}_{k}\right|}{\left|P_{k}\cup G_{k}\right|}$$

where $P_{k}$ is the set of points predicted as class k, and $G_{k}$ is the ground-truth set for class k. Mean IoU (mIoU), which averages the IoUs across all semantic parts, provides a balanced measure of segmentation quality regardless of class imbalance. It provides a balanced measure by averaging the IoUs over all classes:

(12) $$mIoU=\frac{1}{K}\sum_{k=1}^{K}IoU_{k}$$

where K is the total number of semantic parts (in our case, K = 2: Trunk and Crown). These metrics are standard benchmarks for evaluating 3D semantic and part segmentation models [27,28,29,30]. For short-term dendrometer-derived stem-response prediction, we evaluate the regression models using three complementary metrics:

Root Mean Squared Error (RMSE)

The square root of the average squared prediction error:

(13) $$RMSE=\sqrt{\frac{1}{N}\sum_{i=1}^{N}{\left(y_{i}-{\hat{y}}_{i}\right)}^{2}}$$

where $y_{i}$ is observed short-term dendrometer-derived target, ${\hat{y}}_{i}$ is the corresponding predicted target, and N is the number of samples. RMSE penalizes large errors quadratically, making it sensitive to outliers. Lower RMSE indicates better prediction accuracy.

Mean Absolute Error (MAE)

The average absolute prediction error:

(14) $$MAE=\frac{1}{N}\sum_{i=1}^{N}\left|y_{i}-{\hat{y}}_{i}\right|$$

MAE treats all errors equally, regardless of magnitude, providing a more robust metric when outliers are present. It is interpretable in the same biomass-equivalent units as the defined short-term dendrometer-derived target.

Coefficient of Determination ($R^{2}$)

The proportion of variance in the target variable explained by the model:

(15) $$R^{2}=1-\frac{\sum_{i=1}^{N}{\left(y_{i}-{\hat{y}}_{i}\right)}^{2}}{\sum_{i=1}^{N}{\left(y_{i}-\bar{y}\right)}^{2}}$$

where $\bar{y}$ is the mean of true values. $R^{2}$ ranges from −∞ to 1, with 1 indicating perfect prediction and 0 indicating that the model performs no better than predicting the mean. Negative $R^{2}$ values indicate worse-than-baseline performance.

Inference Time

The average time in milliseconds (ms) required to process a single sample on CPU:

(16) $$Inference\;Time=\frac{Total\;Time\;for\;N\;Samples}{N}$$

Inference time was measured on an AMD Ryzen 5 7500F CPU without GPU acceleration as an ×86 CPU computational-efficiency benchmark. INT8 models are expected to run faster than their FP32 counterparts because the fbgemm backend included in PyTorch 2.5.1 used for quantized inference is optimized for integer arithmetic rather than floating point operations. Together, these metrics—accuracy and mIoU for the point-cloud tasks, R^2^, RMSE, and MAE for regression, and CPU inference time for computational efficiency—allow us to evaluate both predictive performance and the computational characteristics relevant to future resource-constrained implementation.

## 4. Experimental Results

Our experimental evaluation embodies a holistic assessment philosophy that transcends narrow metric optimization. Rather than pursuing state-of-the-art benchmarks in isolation, we examine the multi-dimensional trade-space encompassing accuracy, efficiency, interpretability, and deployment feasibility. This comprehensive perspective recognizes that scientific progress in applied machine learning requires not only algorithmic innovation but also rigorous validation under realistic constraints. Our experiments systematically address three fundamental questions: Can deep learning models achieve sufficient accuracy for practical forestry applications? Can quantization maintain this accuracy while reducing computational cost for future edge-oriented implementation? Can multi-modal fusion leverage complementary information sources to surpass single-modality approaches? The experimental design reflects our commitment to reproducibility, transparency, and practical utility, reporting both successes and limitations to guide future research and real-world implementation.

### 4.1. Experimental Setup

To ensure a fair and reproducible comparison between the baseline full-precision models and their quantized counterparts, all experiments were conducted under a standardized and consistent setup. The hardware platform for this study was a high-performance workstation equipped with a 6-core/12-thread AMD Ryzen 5 7500F central processing unit (CPU) (AMD Inc., Santa Clara, CA, USA), 31.1 GB of DDR4 RAM, and an NVIDIA GeForce RTX 4060 Ti graphics processing unit (GPU) (NVIDIA Corporation, Santa Clara, CA, USA) with 16 GB of dedicated GDDR6 VRAM. All procedures were run on an Ubuntu 22.04.5 LTS operating system. The software foundation for our work was the PyTorch deep learning framework, specifically version 2.5.1 compiled with CUDA 12.1, which was managed within a Conda 4.12.0 virtual environment running Python 3.9.23. The complete setup is summarized in Table 6.

For the Quantization-Aware Training (QAT) experiments, PyTorch 2.5.1 (Meta AI, Menlo Park, CA, USA)’s native torch.quantization module with the fbgemm backend included in PyTorch 2.5.1, a choice motivated by its high optimization for ×86 CPU architectures. A consistent set of hyperparameters was maintained across all training runs to facilitate a direct and unbiased comparison. The baseline full-precision (FP32) models were trained on the NVIDIA RTX 4060 Ti GPU to leverage its parallel processing capabilities, while the QAT fine-tuning was performed on the AMD Ryzen 5 CPU in the present implementation to align model optimization with the selected ×86 fbgemm inference backend. For both training scenarios, the Adam optimizer was employed with an initial learning rate of 0.001. The learning rate was dynamically adjusted using a StepLR scheduler, which decayed the rate by a factor of 0.7 every 20 epochs. To mitigate overfitting, a weight decay of 1 × 10^−4^ was applied. All models were trained for a total of 400 epochs. Due to the different memory constraints of the processing units, the batch size for GPU-based FP32 training was set to 16, while a more conservative batch size of 8 was used for CPU-based QAT. The performance of the models was assessed using a suite of standard evaluation metrics. For the classification task, we measured Overall Accuracy (also known as Instance Accuracy) and Mean Class Accuracy. For the part segmentation task, we evaluated the Overall (point-wise) Accuracy and, more importantly, the Mean Intersection over Union (mIoU), which is the primary metric for this task. To quantify the benefits of optimization, we also measured two key efficiency metrics: the Model Size in megabytes (MB) based on the saved checkpoint file size, and the average Inference Time in milliseconds (ms) required to process a single sample on the CPU. The reported latency corresponds to single-model inference on the ×86 CPU and should not be interpreted as end-to-end system latency. Point-cloud acquisition, resampling, geometric feature extraction, environmental-sensor acquisition, regression-stage execution, data transfer, and wireless communication overhead were not included in this timing measurement.

### 4.2. Part Segmentation Performance

**Figure 9:**
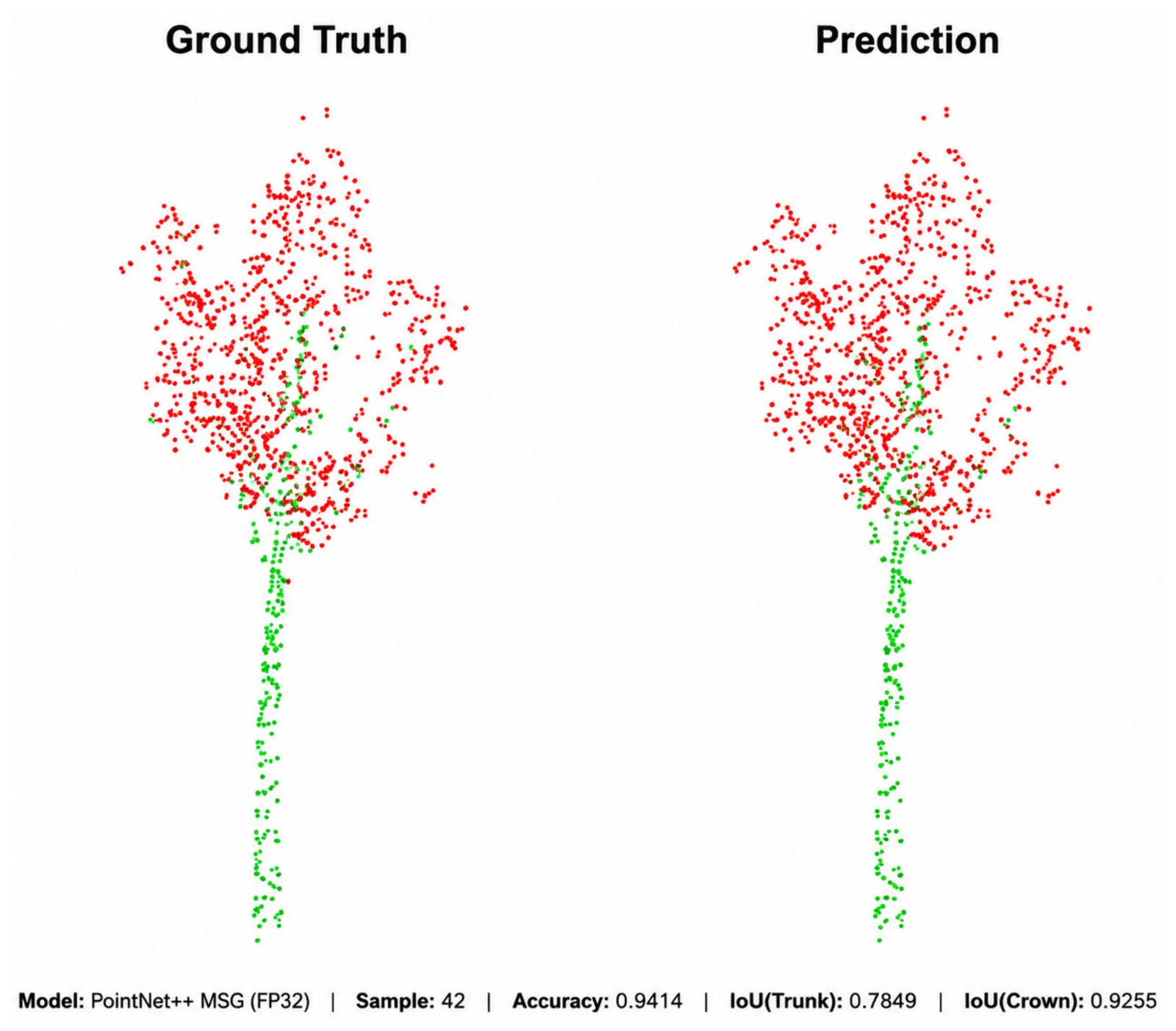
Segmentation results of the FP32 PointNet++ MSG model.

The segmentation task labels each point in a tree’s point cloud as either trunk or crown, which supports the subsequent extraction of sparse-cloud structural descriptors associated with the trunk and crown, including a DBH-related proxy and crown-derived geometric variables [4]. The FP32 baseline, built on the PointNet++ architecture described in Section 3.2, was trained for 400 epochs on the training split. Training loss decreased smoothly from an initial value of about 0.45 at epoch 1 to roughly 0.08 by epoch 400, and validation loss followed a similar path, stabilizing near 0.10 after around 300 epochs without diverging from the training curve. Training accuracy rose from about 85% early on to over 96% by the final epoch, while validation accuracy plateaued near 94% after roughly epoch 250. The close alignment between training and validation curves throughout indicates the model capacity was well matched to the task, and that regularization choices such as weight decay and dropout in the feature propagation layers were effective at preventing overfitting [26]. A representative qualitative example is shown in Figure 9.

On the held-out test set of 270 trees (275,520 points total, since each tree contributes 1024 points), the FP32 model reached 94.14% overall point-wise accuracy and 85.52% mean Intersection over Union (mIoU), the primary metric for this task [4]. Broken down by class, crown segmentation reached 92.55% IoU while trunk segmentation reached only 78.49% IoU. This gap is expected rather than a modeling failure: crown points make up roughly 68% of all points in the dataset, giving the network far more training signal for that class, while trunk regions are geometrically thinner and more frequently occluded by low branches. Detailed FP32 baseline metrics are reported in Table 7.

Visual inspection of individual test trees shows the FP32 model captures overall tree structure with generally clean trunk–crown boundaries: trunk points are correctly identified from the base up to the first major branching point, while crown points correctly cover the rest of the canopy. Subtle errors mostly occur in transition zones where small branches connect trunk to crown, since these regions blend cylindrical, trunk-like geometry with branching, crown-like geometry. Rarer failure modes include multi-stemmed trees with several trunks from a common base, where the model becomes uncertain about where “trunk” ends; heavily pruned trees with sparse crowns, where the model tends to under-segment the crown; and trees with dense, low-hanging branches that occlude the trunk, causing the model to over-extend crown labels into what is actually trunk. Despite these edge cases, overall segmentation quality remains strong, with mIoU above 85%, competitive with other point-cloud segmentation approaches reported in the literature [4,25].

**Figure 10:**
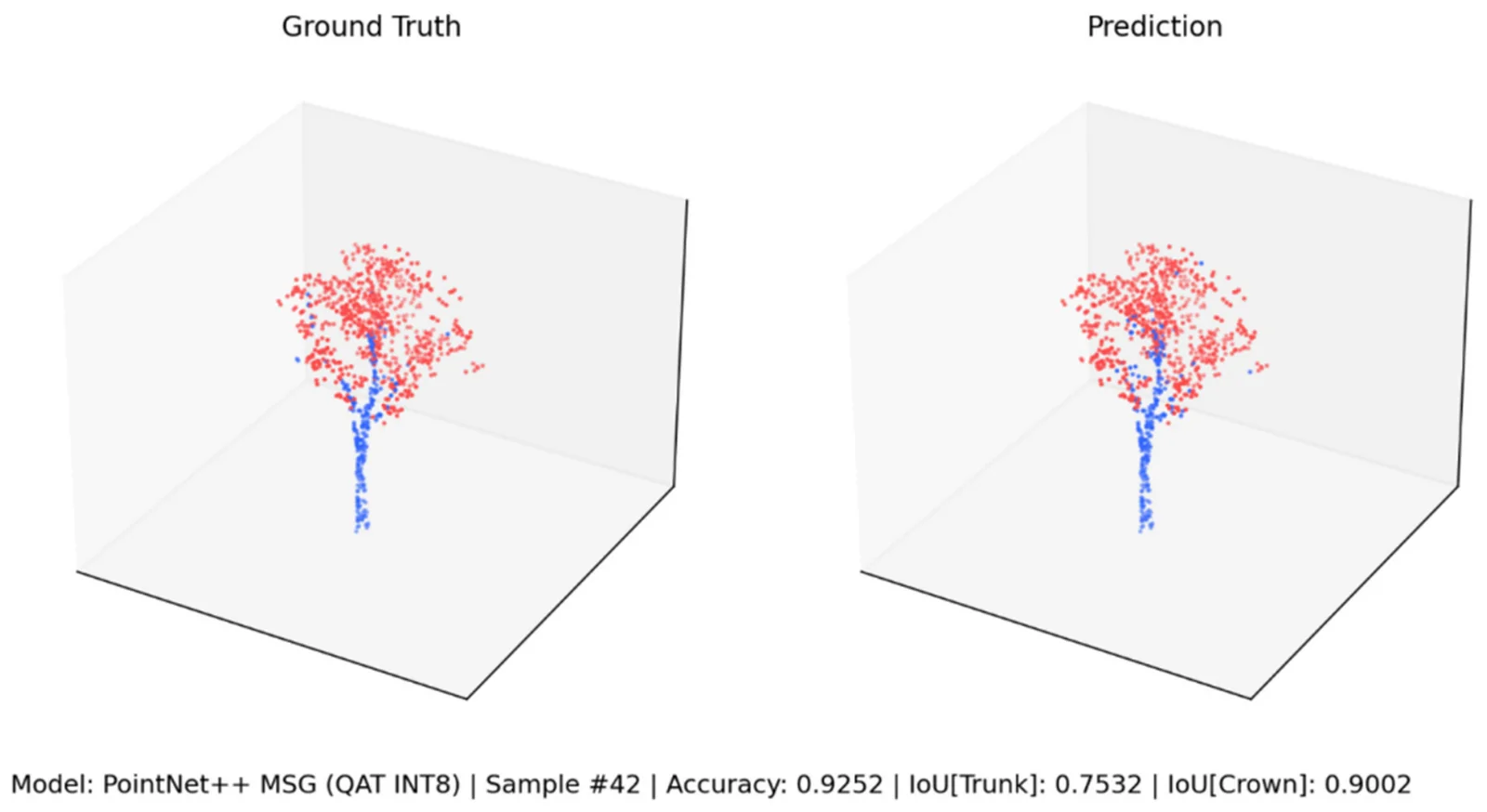
Segmentation results of INT8 (QAT) PointNet++ MSG model.

The FP32 model was then converted to INT8 precision using the Quantization-Aware Training procedure described in Section 3.3 [5]. QAT training showed slightly noisier loss curves than FP32 training, which is expected since fake quantization operations introduce additional noise into the forward pass; training loss dropped from about 0.50 at epoch 1 to 0.11 by epoch 400, while validation loss stabilized near 0.13, a marginally larger training–validation gap than in the FP32 run, suggesting quantization has a mild additional regularizing effect that slightly limits model capacity [26,27]. Validation accuracy reached approximately 92.5% by epoch 350 and remained stable thereafter. The corresponding INT8 result is shown in Figure 10.

Table 8 summarizes the corresponding FP32-versus-INT8 comparison.

The mIoU decreased by 2.85 percentage points, corresponding to a 3.3% relative decrease from the FP32 baseline. The saved checkpoint size decreased from 21.0 MB to 2.0 MB in the present implementation. Because checkpoint size depends on serialization details in addition to numerical precision, this observed reduction should not be interpreted as the theoretical compression ratio of INT8 quantization alone. At the sample level, approximately 70% of test trees showed an mIoU decrease of less than 3 percentage points after quantization, whereas roughly 15% showed a decrease greater than 5 percentage points. Overall, the observed segmentation degradation after quantization was limited in magnitude, although the present analysis does not identify the specific internal representations responsible for this behavior. The results indicate that the QAT-optimized model preserved most of the segmentation capability of the FP32 baseline despite the reduction in saved model size and inference latency. More detailed layer-wise sensitivity analysis would be required to determine which representations were most affected by quantization [30,31,32,33,34,35].

### 4.3. Species Classification Performance

**Figure 11:**
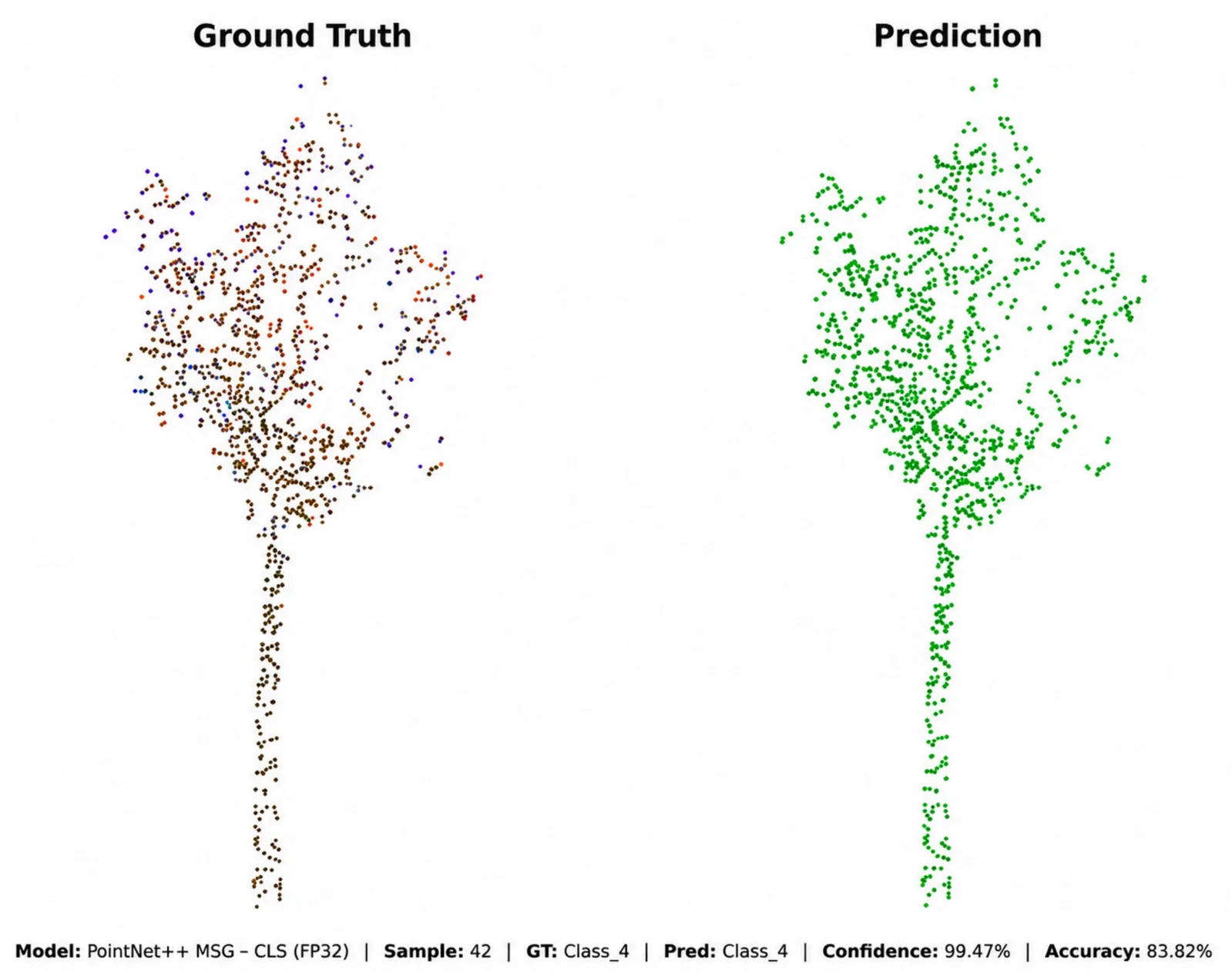
Classification result (FP32 model).

The classification task assigns each tree to one of 34 species, providing taxonomic information that complements the geometric and environmental variables used in the downstream short-term dendrometer-derived stem-response prediction [3,4]. The FP32 classification model converged over 400 epochs, with training loss decreasing from 1.80 to 0.42 and validation loss stabilizing near 0.65. Training accuracy increased from about 35% to 88%, while validation accuracy plateaued around 82% after approximately epoch 250, indicating a persistent train–validation performance gap. A representative result is shown in Figure 11.

On the test set, the FP32 model reached 81.82% overall instance accuracy (221 of 270 trees correctly classified) and approximately 78% mean class accuracy, indicating reasonably balanced performance across species rather than success driven mainly by a few common classes [3]. Full baseline metrics appear in Table 9.

Per-species analysis showed that morphologically distinctive species, such as Metasequoia glyptostroboides with its conical shape, Ginkgo biloba with its fan-shaped leaves, and Pinus densiflora with its needle clusters, were classified with over 90% accuracy, while species within the same genus, for example different Acer (maple) or Quercus (oak) varieties, were frequently confused with each other at only 60 to 70% accuracy, since their point-cloud silhouettes are geometrically very similar even though a botanist could often tell them apart using leaf or bark detail that coarse LiDAR does not fully capture. The 34 × 34 confusion matrix shows systematic, botanically sensible confusions, for instance between Acer palmatum and Acer buergerianum, which mainly differ in leaf size, and between Prunus × yedoensis and Prunus serrulata, which have similar branching patterns. This pattern is consistent with known plant taxonomy rather than a modeling artifact, and indicates the network is learning genuine shape priors, distinctive features such as weeping branches, columnar crowns, or multi-stem growth are classified with high confidence, while generic rounded crowns remain harder to separate [3].

**Figure 12:**
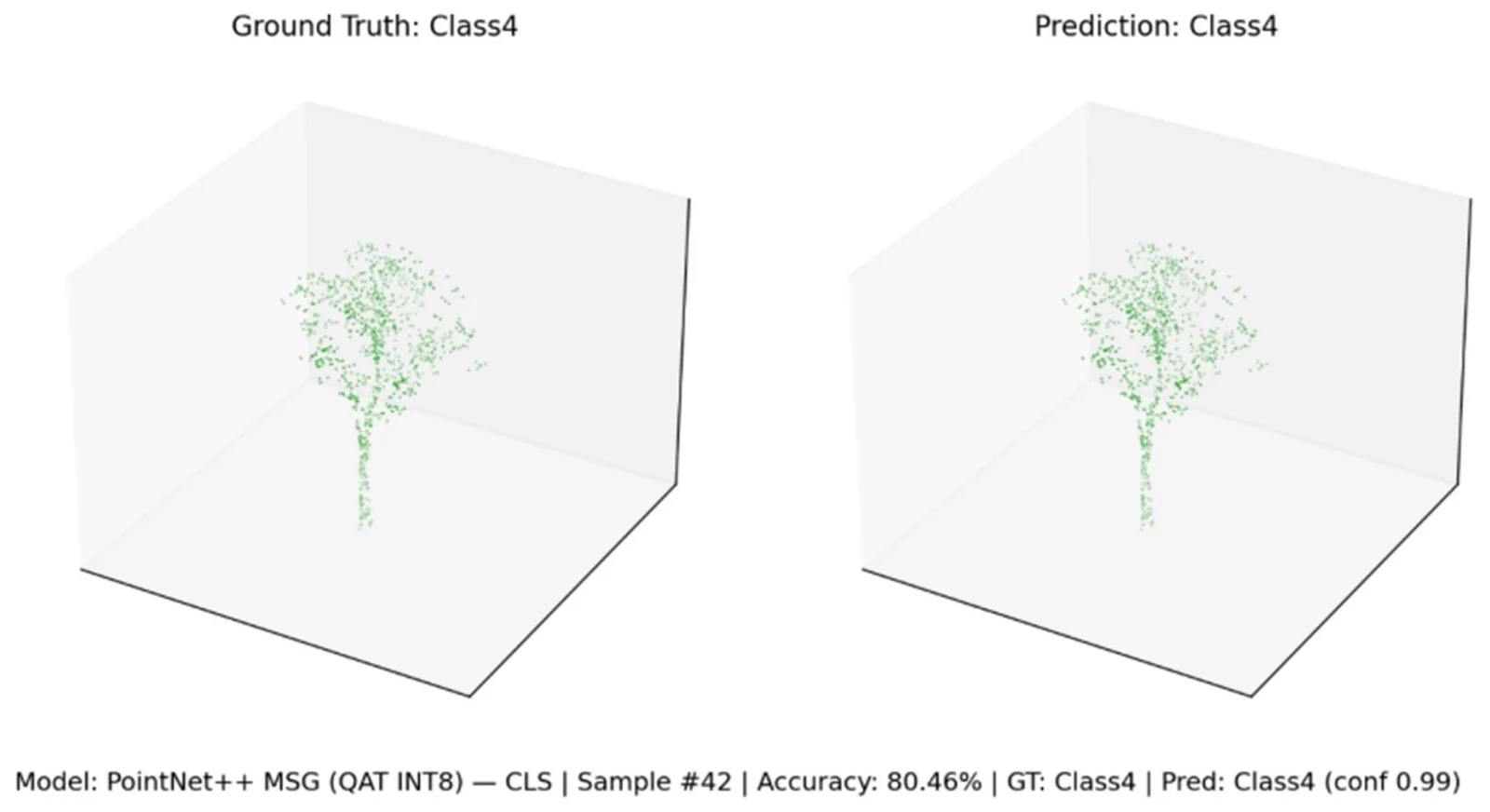
Visualization of classification result (QAT model).

The classification model was then converted to INT8 using the same QAT procedure, trained for 400 epochs on CPU. Training loss decreased from about 2.00 at epoch 1 to 0.55 by epoch 400, while validation loss stabilized near 0.75, slightly higher final losses than the FP32 run, again indicating the capacity constraint imposed by quantization [5]. Validation accuracy reached about 80.5% by epoch 350 and oscillated between 79 and 81% in the final epochs, showing convergence with only minor quantization noise. The corresponding INT8 result is shown in Figure 12.

Table 10 reports the corresponding FP32-versus-INT8 comparison.

The quantized classification model lost only 1.36 percentage points of accuracy while shrinking 10.5-fold and running 4.1 times faster, a slightly better speedup than the segmentation model achieved. Its confusion matrix closely mirrored that of the FP32 model, with no obvious new concentration of errors toward a particular species class after quantization. Rarer species (fewer than 20 samples) showed a somewhat larger accuracy drop, around 2.5 percentage points, than common species (more than 100 samples), around 1.0 percentage point, although the rarer-species accuracy remained above 65% in the present test set [5]. The segmentation and classification tasks showed decreases of 2.85 percentage points in mIoU and 1.36 percentage points in accuracy, respectively; because these metrics quantify different tasks, the magnitude of the drops should not be interpreted as a direct ranking of quantization robustness. Taken together, these results show that quantization reduced the reported task-specific performance metrics by less than 3 percentage points while delivering roughly 10.5× smaller saved checkpoints and up to 4.1× faster ×86 CPU inference, supporting the computational efficiency of the proposed edge-oriented framework [5,27].

### 4.4. Short-Term Dendrometer-Derived Stem-Response Prediction Using MLP and XGBoost

The regression target represented the short-term dendrometer-derived stem response between 8 September 2022 and 22 September 2022, expressed in biomass-equivalent units according to the dataset representation. The frozen classification and segmentation modules were used to generate the taxonomic and structural inputs described in Section 3.4. MLP and XGBoost models were then trained independently using the same 48-dimensional representation and the common 2155/269/270 train, validation, and test split.

**Figure 13:**
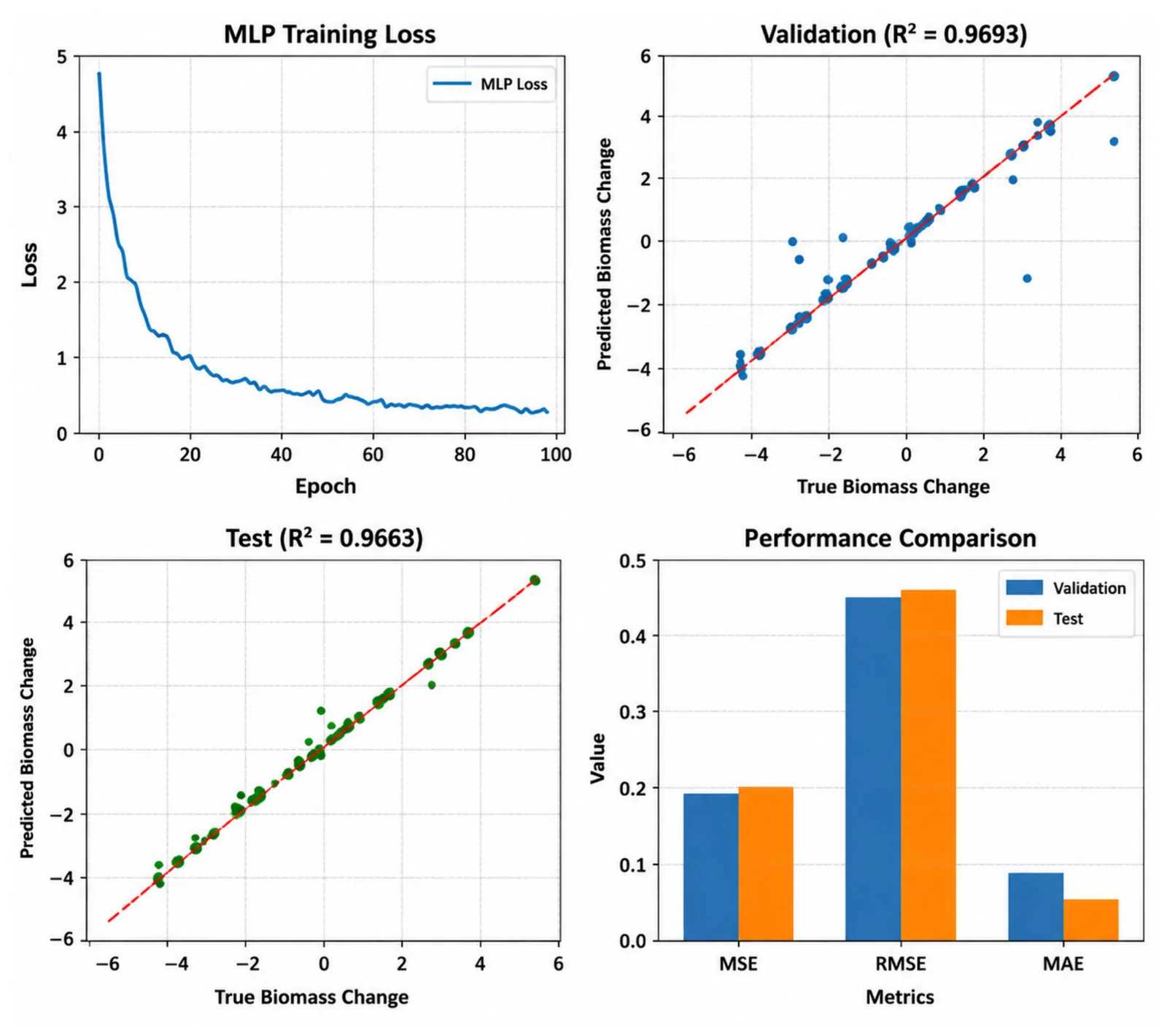
Fusion model training results. (Blue: validation set; Orange: test set; Green: test set in predicted vs. true biomass change plot; Red dashed line: ideal line (y = x)).

With both encoders trained and quantized, their frozen features, combined with the geometric and environmental sensor features described in Table 4, were used to train the two regression heads described in Section 3.4. The MLP head was trained for 100 epochs using the Adam optimizer (learning rate 0.001), on the same 2155/269/270 train/validation/test split used throughout. Training loss dropped sharply within the first 20 epochs and then remained relatively stable for the remainder of training, indicating convergence under the selected training configuration. Training curves are shown in Figure 13.

**Figure 14:**
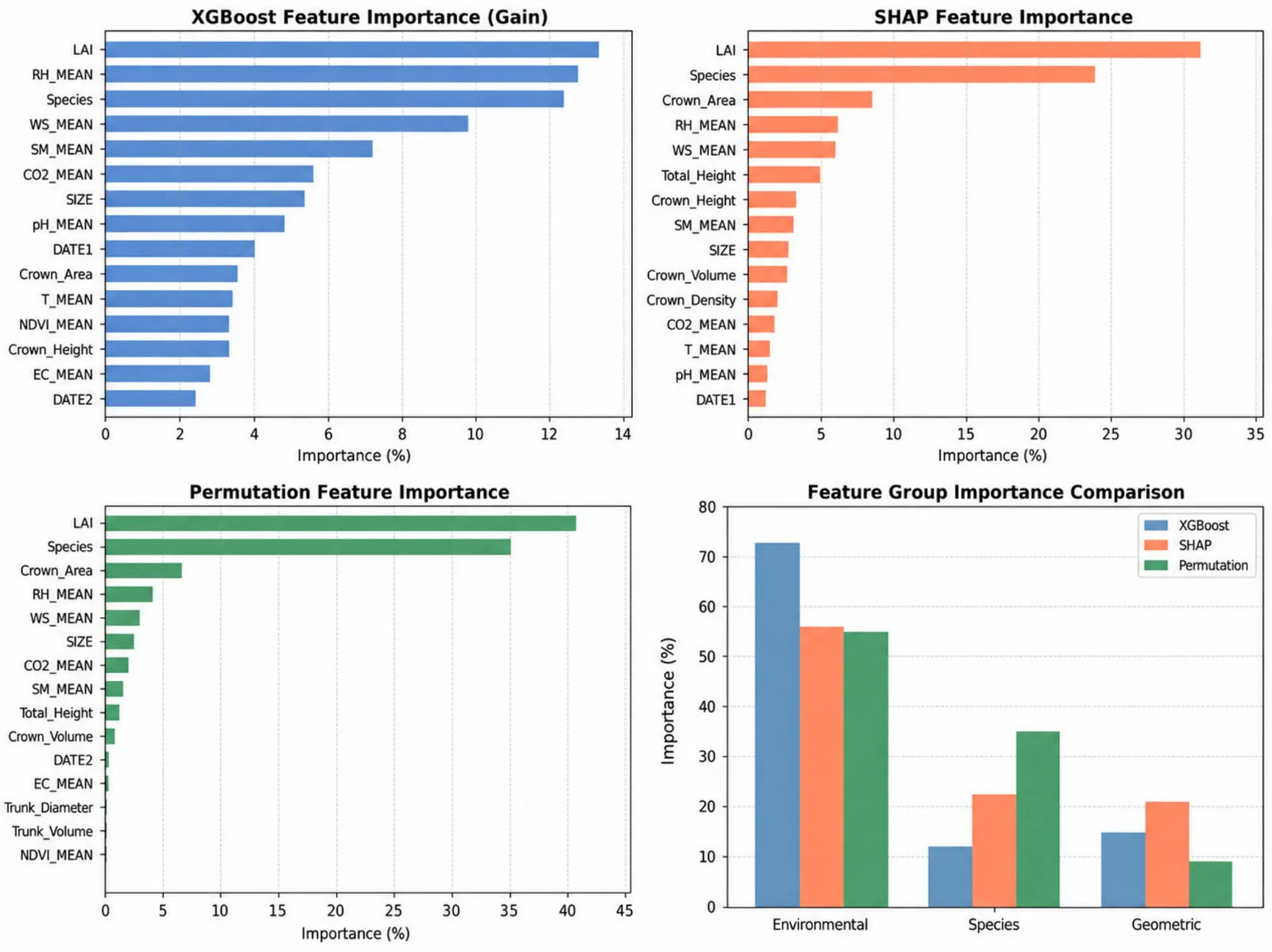
Environmental and geometric features importance.

On the validation set the MLP reached RMSE = 0.4387, MAE = 0.0887, R^2^ = 0.9693, and on the held-out test set, it reached RMSE = 0.4437, MAE = 0.0508, R^2^ = 0.9663. Because a high R^2^ alone cannot establish that the model learned a complex or novel ecological relationship, we additionally evaluated simple structural-size baselines to test whether the prediction target could be explained by an approximately linear relationship with current tree size. A DBH-proxy-only ordinary linear regression achieved R^2^ = 0.0043 on the held-out test set. A tree-height-only regression achieved R^2^ = −0.0091, while a two-variable linear model using both DBH and tree height achieved R^2^ = −0.0011. The test-set Pearson correlations were also weak for DBH (r = −0.0797) and tree height (r = 0.0529), as were the corresponding Spearman correlations (ρ = −0.1030 and ρ = 0.0204, respectively). These diagnostic results therefore provide no evidence that the reported predictive performance can be reduced to a simple linear or monotonic relationship with baseline DBH or tree height. The near-zero performance of the DBH-only baseline also indicates that the full-model accuracy is not attributable to the precision or standalone predictive strength of the sparse-cloud DBH descriptor. Nevertheless, these analyses do not exclude more complex nonlinear structural associations; accordingly, the high full-model R^2^ is interpreted as predictive performance within the present dataset rather than, by itself, as evidence of a novel ecological relationship. The point-density sensitivity analysis also confirmed the limitation of extracting a DBH-related descriptor from the 1024-point representation. With a ±0.05 m diagnostic band centered at breast height, the median number of segmented trunk points was only five, and 17.9% of trees contained fewer than two usable points. Increasing the band width improved point availability, but the maximum-pairwise-distance estimate became increasingly susceptible to isolated or non-stem points. Therefore, the DBH-related input is interpreted as a sparse structural proxy rather than as a validated field DBH measurement. The XGBoost head, trained with 200 estimators on the same fused feature set, allows a direct look at feature importance, shown in Table 11 below. These results are visualized in Figure 14.

These results are visualized in Figure 14.

Species-related variables occupied six of the ten highest-ranked positions in the XGBoost importance analysis. Crown density, tree height, and mean temperature were the most influential non-species variables. These results indicate that the model relied on a combination of taxonomic, structural, and environmental information. Feature importance reflects association with the fitted predictions and should not be interpreted as evidence of causal ecological effects. Among the purely geometric and environmental features, crown density (f3, 50.02) showed the highest feature importance, followed by tree height (f5, 14.96) and mean temperature (f6, 14.74), indicating that structural and environmental variables were associated with the model’s short-term dendrometer-derived response predictions after species information was included. The wide spread of importance scores, from 7.59 to 61.55, indicates that the fitted model relied on geometric, species-related, and environmental variables to substantially different degrees. To assess whether the model relies primarily on species identity rather than genuine per-tree structural variation, we evaluated a naive baseline that predicts each tree’s short-term dendrometer-derived target using only its species-group mean (or region-group mean). This species-only baseline explains just 6.6% of the variance in the short-term dendrometer-derived target (region-only: 13.9%; combined: 15.4%), far below the 96.63% R^2^ achieved by the full model. The substantially lower species- and region-level mean baselines indicate that group identity alone does not reproduce the full-model predictions. However, the absence of overlapping tree IDs does not by itself exclude simple structural associations; this possibility was therefore examined separately using the DBH- and tree-height-based diagnostic regressions described above.

To further quantify the contribution of each feature modality, we conducted an ablation study in which the XGBoost regressor was retrained using different subsets of the 48-dimensional input (Table 12). Among the individual modalities, geometric features alone achieved the highest performance (R^2^ = 0.48), followed by environmental features (R^2^ = 0.35) and species identity alone (R^2^ = 0.30), indicating that static structural measurements carry the strongest standalone signal for predicting short-term biomass-equivalent change, while species and environmental conditions each contribute a smaller, complementary share. Combining modalities consistently improved performance over any single source: Species + Geometric reached R^2^ = 0.58, the strongest two-way combination, ahead of Geometric + Environmental (R^2^ = 0.53) and Species + Environmental (R^2^ = 0.40), showing that geometric information is the most valuable modality to pair with either of the other two. The full XGBoost model using all three feature modalities substantially outperformed every evaluated subset, reaching R^2^ = 0.9645, compared with R^2^ = 0.58 for the strongest two-modality combination. These modality-level results show that the complete feature representation achieved higher predictive performance than the evaluated individual and pairwise feature subsets. However, the ablation results should be interpreted as predictive associations within the present dataset and do not, by themselves, establish a causal ecological mechanism or exclude all possible nonlinear dependencies among structural variables.

Comparing the two regression heads directly (Table 13):

Neither model underwent systematic hyperparameter search; both configurations reflect fixed, reasonable default-like settings for their respective architectures rather than an optimized comparison, so the following performance difference should not be read as establishing one architecture’s inherent superiority. The MLP was slightly more accurate overall and showed lower prediction errors for typical, moderate values of the short-term dendrometer-derived target. The MLP achieved slightly better aggregate test metrics than XGBoost. XGBoost nevertheless provided an interpretable feature-importance analysis and represents a computationally efficient alternative for tabular regression [9,28]. In practice this means the two heads are complementary rather than one simply being better than the other: the MLP is preferable as a general purpose predictor, MLP training is also faster overall, while XGBoost’s built-in feature importance output makes model interpretation easier, and it operates faster at inference time and is more robust on extreme cases, making it a useful complementary predictor, particularly for flagging unusually large short-term dendrometer-derived responses that might warrant closer field inspection [29].

**Figure 15:**
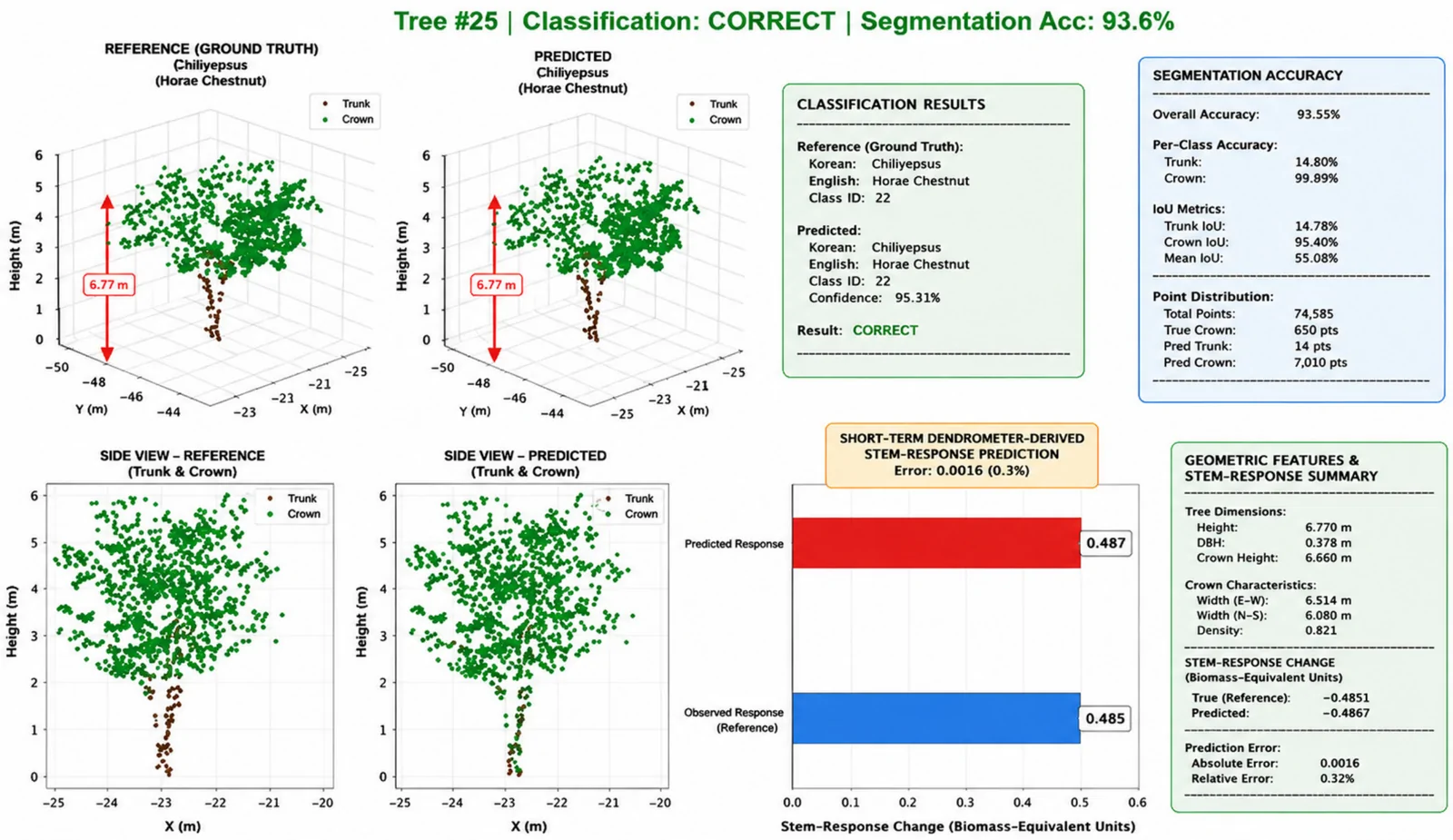
PointNet++ and MLP/XGBoost model visualizations.

This example (Figure 15) for Tree #25 shows reference versus predicted trunk–crown segmentation (93.6% segmentation accuracy for this tree), together with the predicted and observed short-term dendrometer-derived response.

### 4.5. Contextual Comparison with Previously Reported Biomass-Related Prediction Studies

To provide broad methodological context, Table 14 summarizes previously published biomass-related prediction approaches. Because these studies differ from the present work in target definition, observation interval, dataset, and validation protocol, the comparison is descriptive only and does not constitute a direct performance benchmark.

The studies summarized in Table 13 used different datasets, target definitions, observation levels, biomass ranges, sample sizes, and validation protocols. Their reported metrics are therefore presented only as contextual references and should not be interpreted as a direct benchmark ranking. In particular, differences in target variance strongly affect both R^2^ and RMSE. A rigorous model comparison would require retraining all baseline methods on the same dataset and using the same train, validation, and test partitions.

The proposed model achieved R^2^ = 0.9663 on the held-out tree-level test partition. However, because the studies summarized above used different datasets, target definitions, observation intervals, and validation protocols, differences in reported R^2^ values should not be interpreted as evidence that the proposed model is intrinsically more predictive than those approaches. In particular, the high R^2^ obtained in the present dataset does not by itself demonstrate that the model learned a more complex ecological relationship. We therefore treat the literature comparison as contextual only and evaluate the possibility of a simple size-related association using internal diagnostic baselines on the same dataset. Furthermore, because the present split is random at the individual-tree level rather than region-held-out, the reported performance should not be interpreted as evidence of generalization to entirely unseen geographic regions [40].

## 5. Discussion

The proposed framework combines AI-driven point-cloud perception, multimodal feature fusion, and model compression for short-term urban-tree monitoring. Rather than treating perception, compression, and regression as independent computational stages, the present results show that QAT can substantially reduce the storage footprint and ×86 CPU inference latency of the PointNet++ perception models while preserving most of their classification and segmentation performance. These results support computational efficiency for future edge-oriented implementations, but they do not establish the practical reliability of a fully integrated edge sensing device. Species-related variables, crown density, tree height, and mean temperature emerged as influential predictors of the short-term dendrometer-derived target. These associations should be interpreted as relationships with short-term stem dynamics rather than evidence of biological control over irreversible biomass accumulation. Because the observation interval was only 14 days, the target may include substantial reversible diameter variation associated with tree water status in addition to structural growth. Therefore, neither the target values nor the reported regression accuracy should be interpreted as estimates of annual biomass accumulation or carbon sequestration. The unusually high regression R^2^ also warrants cautious interpretation, because predictive fit alone does not demonstrate that the model has learned a novel or complex ecological relationship. To examine the Reviewer’s specific concern regarding a potentially trivial baseline-size effect, simple linear diagnostic models were evaluated using DBH, tree height, and their combination. The resulting R^2^ values were 0.0043 for DBH alone, −0.0091 for tree height alone, and −0.0011 when both size variables were combined. The corresponding Pearson and Spearman correlations were also weak. These results do not support a strong simple linear or monotonic relationship between the short-term target and these baseline measures of tree size. However, they do not exclude more complex nonlinear structural associations. Accordingly, the novelty of the present study is not claimed on the basis of the high R^2^ alone, but primarily from the integration of QAT-compressed point-cloud perception with multimodal downstream prediction. Expressing the short-term dendrometer-derived response as a fraction of each tree’s total standing biomass would require an independent, species-specific allometric estimate of above-ground biomass, which was outside the scope of the present study. We therefore emphasize that the reported RMSE and R^2^ quantify prediction of the defined short-term dendrometer-derived response and should not be interpreted as indicating any fraction of total tree biomass. A specific technical limitation concerns the DBH-related descriptor extracted from the 1024-point representation. The point-density sensitivity analysis showed that very few segmented trunk points may be available close to breast height; within a ±0.05 m diagnostic band centered at 1.3 m, the median was five points and 17.9% of trees contained fewer than two usable points. Moreover, increasing the vertical band improved point availability at the cost of greater sensitivity of the maximum-pairwise-distance heuristic to isolated and non-stem points. We therefore do not interpret this sparse-cloud descriptor as a field-equivalent DBH measurement. Instead, it is retained only as one coarse structural input within the multimodal feature set. This interpretation is also consistent with the DBH-only diagnostic regression, which achieved R^2^ = 0.0043 on the held-out test set and therefore provided essentially no standalone predictive power. Direct validation of sparse-cloud DBH against independent field measurements, together with denser or trunk-focused sampling and more robust circle- or cylinder-fitting methods, is required before this component can be considered reliable for operational forestry inventory applications. The biological interpretation of the dendrometer-derived target is a scope boundary of the present study rather than merely a secondary measurement limitation. Dendrometer observations over a 14-day interval quantify short-term stem-diameter dynamics that may reflect reversible water-status-related expansion and contraction together with any structural change occurring during the same period. They therefore do not provide a biological reference for dry-biomass accumulation. In the present framework, the dendrometer-derived quantity is used only as the independently measured reference response that the regression models are trained to predict. Consequently, the reported regression metrics quantify prediction of this defined short-term sensor-derived response and should not be interpreted as validation of biomass gain, carbon sequestration, or irreversible growth. Establishing such biological interpretations would require independent biomass-reference measurements together with seasonal or multi-year observations. We further note that the current train/validation/test partition is a random tree-level split and does not hold out any of the five surveyed regions entirely; the reported performance therefore demonstrates generalization to unseen individual trees within the sampled regions, but not to geographically distinct, entirely unseen regions. Confirming cross-region generalization would require a region-held-out validation protocol. Such an evaluation was not performed in the present study and is identified as an important direction for future validation [41]. From a computational perspective, the reduction in model size and ×86 CPU inference latency demonstrates that QAT can reduce the resource requirements of the LiDAR perception modules. However, the present study does not demonstrate a fully integrated or field-validated edge sensing system. The experiments were conducted on an ×86 desktop CPU rather than on a low-power embedded platform, and the reported latency represents model inference rather than end-to-end sensing latency. Energy consumption, peak memory usage, point-cloud acquisition and preprocessing time, environmental-sensor integration, regression-stage latency, communication overhead, and long-term field reliability were not measured. In addition, as discussed above, the sparse-cloud DBH-related descriptor requires independent validation before it can support operational forestry measurement. Consequently, the present work should be interpreted as an edge-oriented computational evaluation and proof of model-compression feasibility, not as evidence of completed practical edge deployment [42,43,44].

## 6. Conclusions

This study presented an AI-driven framework for predicting a short-term dendrometer-derived stem response by combining QAT-optimized PointNet++ models with multimodal regression. The dataset consisted of 2694 individual trees from five regions of South Korea, with the prediction target derived from stem-diameter measurements collected over a 14-day interval between 8 September 2022 and 22 September 2022 and represented in biomass-equivalent units. The proposed framework integrated point-cloud perception, geometric analysis, environmental information, and machine-learning regression within a unified processing pipeline. The QAT-optimized PointNet++ models preserved most of the performance of their FP32 counterparts while substantially reducing model size and CPU inference time. The optimized species-classification model achieved 80.46% accuracy across 34 species, while the trunk–crown segmentation model achieved 92.52% overall accuracy and 82.67% mIoU. These results show that quantization can improve computational efficiency without causing a major loss of predictive capability. For short-term stem-response prediction, the framework combined a 34-dimensional predicted species representation, six geometric variables, and eight environmental and vegetation-related variables into a 48-dimensional input vector. The regression stage achieved strong predictive performance, with the MLP obtaining R^2^ = 0.9663, RMSE = 0.4437 kg, and MAE = 0.0508 kg, while XGBoost achieved R^2^ = 0.9645, RMSE = 0.4510 kg, and MAE = 0.0532 kg. These high R^2^ values characterize predictive fit within the present randomly partitioned dataset and should not, by themselves, be interpreted as evidence of a novel ecological mechanism or of generalization to entirely unseen regions. Feature-importance analysis further indicated that species-related variables, crown density, tree height, and mean temperature were among the most influential predictors. From the perspective of intelligent sensors and IoT systems, the proposed workflow demonstrates how raw LiDAR data can be transformed into compact semantic and predictive outputs through automated classification, segmentation, feature extraction, and regression. This approach can reduce the need to transmit full point clouds and may support future distributed urban-forest monitoring platforms in which sensing devices perform part of the analysis locally before communicating results to central systems. However, the present work should be considered an edge-oriented computational evaluation rather than a completed embedded deployment. The experiments were conducted on an ×86 desktop CPU, and the dendrometer-derived prediction target covered only a 14-day observation period. Future work should validate the framework on representative low-power embedded platforms by measuring end-to-end latency, peak memory use, energy consumption, sensor integration, communication overhead, and field reliability, while also evaluating generalization using a region-held-out validation protocol. Future validation should also quantify the accuracy and repeatability of sparse-cloud structural descriptors, particularly the DBH-related proxy, against independent field measurements and under different point-density conditions. Most importantly, the present 14-day target should not be interpreted as a direct measure of dry-biomass accumulation. The reported regression results demonstrate prediction of the defined short-term dendrometer-derived response only. Establishing biological validity for biomass accumulation or carbon sequestration will require independent biomass-reference measurements and seasonal or multi-year observations. Extending the dataset to seasonal and multi-year observation periods will also be necessary before the framework can support long-term growth analysis or carbon-sequestration monitoring.

## Figures and Tables

**Table 1:** Shared PointNet++ encoder configuration, with classification-specific and segmentation-specific heads.

Module	Layer	Input → Output	Configuration	Parameters
Shared encoder	SA1	1024 × 6 → 512 × 128	r = 0.2 m, K = 32, MLP [64, 64, 128]	~50 K
Shared encoder	SA2	512 × 128 → 128 × 256	r = 0.4 m, K = 64, MLP [128, 128, 256]	180 K
Shared encoder	SA3	128 × 256 → 32 × 512	r = 0.8 m, K = 128, MLP [256, 256, 512]	721 K
Classification head	SA4	32 × 512 → 1 × 1024	Global pooling, MLP [256, 512, 1024]	1.05 M
Classification head	FC1–FC3	1024 → 512 → 256 → 34	Linear + BN + ReLU + Dropout, softmax	665 K
Segmentation head	FP3	32 × 512 + 128 × 256 → 128 × 256	IDW interpolation + skip	395 K
Segmentation head	FP2	128 × 256 + 512 × 128 → 512 × 128	IDW interpolation + skip	164 K
Segmentation head	FP1	512 × 128 + 1024 × 6 → 1024 × 64	IDW interpolation + skip	33 K
Segmentation head	Head	1024 × 64 → 1024 × C	1 × 1 Conv, softmax	4.2 K

**Table 2:** QAT configuration used to convert both encoders from FP32 to INT8.

Component	Configuration	Description
Weight quantization	INT8, symmetric, per-channel	Range [−127, 127], zero-point = 0
Activation quantization	INT8, asymmetric, per-tensor	Range [0, 255], learned zero-point
Calibration dataset	1000 training samples	Used for scale initialization
QAT learning rate	0.001 (same as FP32 rate)	Fine-tuning rate after quantization
Optimizer	Adam (β1 = 0.9, β2 = 0.999)	Same optimizer as the FP32 baseline
Batch size	24 (classification), 16 (segmentation)	Same as FP32 training
Backend	PyTorch Quantization API, fbgemm	Inserts FakeQuantize modules
Epochs	400	Same schedule as FP32 baseline

**Table 3:** Sensitivity of breast-height trunk-point availability to the diagnostic vertical-band width in the 1024-point representation. Note: The band-width analysis is a post hoc sensitivity test intended to quantify point availability near breast height; it does not redefine the regression input used in the original experiments.

Half-Band Around 1.3 m	Median Trunk Points	Trees with <2 Trunk Points
±0.025 m	2	37.9%
±0.050 m	5	17.9%
±0.075 m	7	10.5%
±0.100 m	10	7.2%
±0.150 m	15	3.5%
±0.200 m	20	2.3%

**Table 4:** Feature sources and physical categories feeding the fusion stage.

Source	Category	Features	Dim
Classification model	Species	One-hot species encoding	34
Segmentation model	Geometric	DBH-related proxy, crown width (E–W, N–S), crown density, crown/tree height	6
Environmental sensors	Climate	Temperature, CO_2_, humidity	3
Environmental sensors	Soil	pH, electrical conductivity	2
Auxiliary dataset variables	Vegetation-related	NDVI, LAI, SIZE (processed dataset field)	3
Total		Multi-modal fusion input	48

**Table 5:** Regression head configurations: MLP and XGBoost.

Head	Configuration	Rationale
MLP	3 hidden layers [256, 128, 64] BN + ReLU each 128-dim embedding output = 1	Smooth, stable predictions across the bulk of the data distribution
XGBoost	200 estimators max depth = 6 learning rate η = 0.1 subsample = 0.8 colsample = 0.8 L1 = 0.1 L2 = 1.0 gamma = 0.1 min_child_weight = 3	Robust to outliers and extreme short-term target values

**Table 6:** Standardized experimental setup of FP32 vs. QAT models.

Components	Specification
Hardware	CPU: AMD Ryzen 5 7500F (6 cores, 12 threads), GPU: NVIDIA RTX 4060 Ti (16 GB)
Software	Software: Python 3.9.23 (Python Software Foundation, Wilmington, DE, USA), PyTorch 2.5.1 (Meta AI, Menlo Park, CA, USA), CUDA Toolkit 12.1 (NVIDIA Corporation, Santa Clara, CA, USA).
Quantization	QAT with torch.quantization (fbgemm backend included in PyTorch 2.5.1)
Training	Optimizer: Adam (LR = 0.001), Epochs: 400
FP32 Training	GPU, batch size = 16
QAT Training	CPU: AMD Ryzen 5 7500F (6 cores, 12 threads)
Classification Metrics	Overall Accuracy, Mean Class Accuracy
Segmentation Metrics	Overall Accuracy, Mean IoU
Efficiency Metrics	Model Size (MB), Inference Time (ms)

**Table 7:** Part segmentation performance, FP32 baseline.

Metrics	Value
Overall Accuracy (Point-wise)	94.14%
Mean IoU (mIoU)	85.52%
IoU (Trunk)	78.49%
IoU (Crown)	92.55%
Model Size	21.0 MB
Inference Time (CPU)	27.71 ms

**Table 8:** Part segmentation performance, FP32 versus INT8.

Metric	FP32	INT8 (QAT)	Change
Overall Accuracy	94.14%	92.52%	−1.62 pp (−1.72%)
Mean IoU (mIoU)	85.52%	82.67%	−2.85 pp (−3.33%)
IoU (Trunk)	78.49%	75.32%	−3.17 pp (−4.04)
IoU (Crown)	92.55%	90.02%	−2.53 pp (−2.73)
Model Size	21.0 MB	2.0 MB	10.5× smaller
Inference Time	27.71 ms	7.016 ms	3.95× faster

**Table 9:** Species classification performance, FP32 baseline.

Metric	Value
Overall Accuracy (Instance)	81.82%
Mean Class Accuracy	~78%
Prediction Confidence (Sample)	99.47%
Model Size	21.1 MB
Inference Time (CPU)	26.337 ms

**Table 10:** Species classification performance, FP32 versus INT8.

Metric	FP32 Baseline	QAT INT8	Change
Overall Accuracy	81.82%	80.46%	−1.36 pp (−1.66%)
Mean Class Accuracy	78%	76%	−2 pp (−2.56%)
Confidence (Sample)	99.47%	99%	−0.47 pp (−0.47%)
Model Size	21.1 MB	2.0 MB	10.5× smaller
Inference Time	26.337 ms	6.423 ms	4.1× faster

**Table 11:** Top features by XGBoost importance score.

Feature	Description	Importance
f16	Species class (id × 2)	61.55
f3	Crown Density	50.02
f19	Species class (id × 5)	44.66
f14	Species class (id × 0)	38.62
f27	Species class (id × 13)	16.54
f5	Tree Height	14.96
f6	Env. feature (T_MEAN)	14.74
f22	Species class (id × 8)	13.91
f15	Species class (id × 1)	12.68
f30	Species class (id × 16)	7.59

**Table 12:** Diagnostic baseline and feature-ablation results for short-term target prediction on the held-out test set.

Configuration	Model	Dimensions	R^2^
DBH only	Linear Regression	1	0.0043
Tree height only	Linear Regression	1	−0.0091
DBH + tree height	Linear Regression	2	−0.0011
Species only	XGBoost	34	0.30
Geometric only	XGBoost	6	0.48
Environmental only	XGBoost	8	0.35
Species + Geometric	XGBoost	40	0.58
Species + Environmental	XGBoost	42	0.40
Geometric + Environmental	XGBoost	14	0.53

**Table 13:** MLP and XGBoost model performances.

Model	RMSE (kg)	MAE (kg)	R^2^
MLP	0.4437	0.0508	0.9663
XGBoost	0.4510	0.0532	0.9645

**Table 14:** Contextual comparison with previously published biomass-related prediction studies.

Models	Reported R^2^	Reported RMSE
SSAE (NEW) [10]	0.935	0.1567
PointNeXt [11]	0.920	0.18
Random Forest [35]	0.873	0.793
XGBoost [28]	0.868	0.793
Multivariate Linear [36]	0.851	0.3120
Point Transformer V3 [37]	0.823	0.3845
CNN-LSTM (NEW) [38]	0.802	0.2210
DNN Fusion (NEW) [39]	0.770	0.2838
Proposed model	0.9663	0.4437

Note: RMSE and R^2^ values are reported as originally presented in the cited studies. Because the studies used different datasets, target definitions, observation intervals, target variances, and validation protocols, neither metric should be interpreted as directly comparable across studies.

## Data Availability

The data supporting the findings of this study are available from the corresponding author upon reasonable request.
